# Cognitive structure and progression in Parkinson’s disease: insights from a tablet-based assessment

**DOI:** 10.1038/s41598-026-62324-6

**Published:** 2026-07-15

**Authors:** Tim Feige, Anika Frank, Jonas Bendig, Andrea Epler, Charlotte Harbarth, Julia Kunze, Johanna Jungk, Nils Schnalke, Heinz Reichmann, Björn H. Falkenburger

**Affiliations:** 1 Department of Neurology, University Hospital Carl Gustav Carus, Technische Universität Dresden, Fetscherstrasse 74, 01307 Dresden, Germany; 2 German Center for Neurodegenerative Diseases (DZNE), Dresden, Germany

**Keywords:** Parkinson’s disease, Cognition, Digital, Open source, Diseases, Health care, Medical research, Neurology, Neuroscience, Psychology, Psychology

## Abstract

Cognitive impairment is an important constraint for PwPD with Parkinson’s disease (PwPD). Digital assessments potentially provide more accessible measurements and obtain a richer set of data than traditional paper–pencil-based instruments. Open-source generation can support the availability of comparable assessments in different cohorts. A digital, tablet-based cognitive assessment (DiCo) comprising 13 distinct tests was implemented as an open-source tool, expanding on available frameworks. Commonly used tests were selected to cover response inhibition, cognitive flexibility, attention, and working memory. After extensive usability testing, the DiCo was applied in participants without overt cognitive impairment. 97 participants (43% women) completed the entire DiCo. Clustering of DiCo feature correlations and conditional dependencies indicated a predominantly mutual organization of cognitive performance in PwPD. Exploratory factor analysis identified five interrelated latent factors. Most factors were derived from individual tests and correlated moderately with traditional neuropsychological tests and questionnaires. Machine learning identified a test of working memory (N-back) as the most predictive feature for the Montreal Cognitive Assessment. Latent profile analysis revealed four cognitive subgroups, mainly reflecting severity. The open-source digital cognitive assessment established in this study showed good usability by PwPD. The observed overlap between individual DiCo features and MoCA and FAB scores may inform future reduced task sets for research or selected clinical settings.

**Supplementary Information:**

The online version contains supplementary material available at 10.1038/s41598-026-62324-6.

## Introduction

Parkinson’s disease (PD) is the second most common neurodegenerative disease and the fastest growing neurological disorder in the world with regard to prevalence, disability and deaths^1,2^. PD is defined by its cardinal motor symptoms resting tremor, rigidity and bradykinesia. In addition, PD encompasses a multitude of non-motor symptoms (NMS). People with PD (PwPD) have a 2.8 to sixfold higher risk of developing dementia than the general population^3^. Several longitudinal studies have demonstrated that almost all PwPD develop dementia if they live with PD for more than 10 years^4^. Hence, cognitive impairment is one of the most common NMS and arguably the NMS that PwPD fear most^5^.

Cognitive impairment can be classified based on severity and based on the affected functional systems. The following severity levels are currently delineated: normal cognition (NC), Subjective Cognitive Decline (SCD), Mild Cognitive Impairment (MCI), and Parkinson’s Disease Dementia (PDD)^6–8^. In addition, the following functional domains are distinguished by the Movement Disorder Society: attention/working memory, executive functions, language, memory, and visuospatial functions^9^. The timing, profile, and rate of cognitive decline can be highly variable in PD^10^. The observation of distinct functional impairment profiles in MCI has led to the hypothesis of the "dual syndrome," suggesting two subtypes of PD-MCI. These are based on the impairment of distinct anatomical regions: the frontal syndrome, associated with executive and attentional deficits, and the posterior cortical syndrome, associated with visual-spatial and memory deficits^11^.

Digital assessments build on a long tradition of psychophysical research and potentially offer the opportunity to collect an amount of data and a range of features for cognitive function that are beyond of what is possible with classical paper–pencil-based neuropsychological testing (NPT). Hence, digital assessments allow for data driven analyses that can potentially reveal novel patterns and relationships that were not possible before^12^. In addition, digital testing methods have been suggested to allow for low-threshold longitudinal measurements with high detail, applicable with minimal effort in a large population^13,14^. Digital assessments should provide at least the information obtained by commonly used screening instruments such as the Montreal Cognitive Assessment (MoCA)^15^ and the Frontal Assessment Battery (FAB)^16^. Among digital methods, open-source tools support the use of comparable tests in different cohorts as a basis for the generation of poolable datasets for data-driven investigation into cognitive profiles associated with neurodegenerative disorders and the modeling of disease progression trajectories.

We therefore intended to implement a digital, tablet-based cognitive assessment battery (DiCo) covering cognitive domains and impulsivity-related constructs relevant to PD and expanding on readily available, open-source systems and frameworks such as the *Experiment Factory* (EF)^17^ and jsPsych^18^. The selection of tests was based on a systematic review of the literature. Only established assessments or adaptations thereof were included in the implementation. A mixed-method approach to usability testing with PwPD and PD experts was performed to ensure applicability in research and practice.

A second aim was to use the comprehensive digital feature set to investigate cognitive heterogeneity in Parkinson’s disease, specifically whether performance across cognitive and impulsivity-related tasks reveals distinct cognitive profiles. To this end, we applied complementary exploratory analyses to the extracted feature-level data. Hierarchical clustering, network analysis, and exploratory factor analysis were used to examine shared and distinct dimensions of digital cognitive performance, whereas latent profile analysis explored inter-individual performance profiles. Random forest models were additionally trained to assess the degree of overlap between DiCo-derived features and established global cognitive screening measures such as MoCA and FAB. Finally, in a small exploratory longitudinal subset, we examined whether DiCo-derived performance measures showed measurable change over time and whether such changes aligned with clinical progression markers.

## Results

### Study participants

After usability testing, the tablet-based digital assessment was administered to a cohort of *n* = 100 people with Parkinson’s disease (43% women) without severe cognitive impairment, as indicated by MoCA score ≥ 18, of whom 97 completed the entire test battery. The three dropouts had different reasons: Two PwPD had to terminate the study earlier because of scheduled follow-up medical appointments and one felt too exhausted to continue the testing. Demographic and clinical data of all participants are displayed in Table 1. With an average disease duration of 6.4 years and a mean LEDD of 672 mg, our sample is beyond the typical ‘honeymoon period’ of Parkinson’s disease, which is usually defined as the first 2–5 years of stable response to levodopa^19^. The average score in the Montreal Cognitive Assessment (MoCA) was 26 points. 58 (60%) participants classified as cognitively normal and 39 (40%) classified as having mild cognitive impairment based on the established MoCA cutoff score of < 26^15^.

**Table 1:** Demographic and clinical characteristics.

Feature (at baseline)	Entire cohort (n = 97)	Completed paper–pencil tests (= 50)	Completed questionnaires (n = 76)	Follow-up (n = 28)
Gender, % male^(1)^	55 (57%)	29 (58.0%)	41 (53.9%)	13 (46%)
Age (years)^(2)^	63 (10.1), [36–82]	62.6 (9.3), [37–84]	63.4 (9.3), [36–84]	62 (9.8), [36–89]
Disease Duration (years)^(2)^)	6.3 (4.7), [0–26]	5.6 (4.4), [0–19]	6.3 (5.0), [0–26]	6.4 (3.9), [1–15]
Age at onset (years)^(2)^	56 (10.5), [30–82]	56.7 (9.8), [30–82]	56.9 (10.0), [33–82]	56 (10.6), [32–77]
Years of education	10.8 (1.2), [8–13]	11.0 (1.2), [8–13]	10.8 (1.2), [8–13]	11.1 (1.1), [9–13]
LEDD Total (mg)^(2)^	672 (338.8), [0–1620]	620.8 (333.8), [0–1445]	671.9 (355.8), [0–1620]	665 (355.6), [200–1445]
LEDD LDopa (mg)^(2)^	418 (197.7), [0–973]	391.6 (193.5), [0–800]	420.6 (200.2), [0–973]	365 (189.3), [0–775]
LEDD DA (mg)^(2)^	244 (141.6), [0–925]	239.3 (168.3), [26–925]	248.6 (149.2), [30–925]	268.8 (126.8), [52–560]
Neuroleptics^(1)^	6 (6%)	4 (8.0%)	5 (6.6%)	1 (3.5%)
Hallucinations^(1)^	5 (5%)	2 (4.0%)	4 (5.3%)	0
Vascular Risk present^(1)^	34 (35%)	14 (28.0%)	26 (34.2%)	7 (25%)
UPDRS III^(2)^	19 (9.0), [4–48]	19.8 (8.6), [6–42]	18.2 (8.5), [4–42]	17.4 (7.5), [6–36]
MoCA^(2)^	26 (2.8), [18–30]	26.7 (2.4), [21–30]	26.6 (2.8), [18–30]	27.8 (1.8), [24–30]
≥ 26	58 (60%)	31 (62.0%)	48 (63.2%)	24 (86%)
FAB^(2)^	16.5 (1.9), [7–18]	17.1 (1.5), [12–18]	16.5 (1.9), [7–18]	17.4 (0.8), [15–18]
SCD^(1)^	53 (54.7%)	30 (60.0%)	41 (53.9%)	17 (60%)

(1) *N and %; *(2) Mean (SD), [Range]; LEDD—levodopa equivalent daily dose; DA—dopamine agonist; MoCA—montreal cognitive assessment, FAB—frontal assessment battery, SCD—subjective cognitive decline

A convenience subset of n = 28 participants was available for a follow-up assessment. The mean interval between the two assessments was 25 months. The follow-up assessment was not based on a predefined longitudinal retention protocol; rather, participants were reassessed when they were willing and available for repeat testing. Several baseline participants declined repeated participation or were not available for follow-up. Demographics of this subset are also included in Table 1. Compared with the full baseline cohort, the follow-up subset showed numerically higher MoCA and FAB scores, indicating that longitudinal analyses may overrepresent cognitively healthier participants.

Another subset of n = 50 participants additionally took part in a paper–pencil neuropsychological assessment (NPT) that comprised the CERAD+ ^20^. Additional questionnaire (Q-Data) was available for n = 76 participants. An overview of all DiCo tasks, NPT tests and questionnaires is provided in Table 2.

**Table 2:** Feature dictionary.

Feature	Abbreviation	Cognitive domain/construct	Direction (Higher = better)	Retained after VIF	Retained in factor solution after MSA
DiCo					
Simple Reaction^17^	SR				
Mean reaction time	SR—RT-M		−	+	−
Reaction time sd	SR—RT-SD		−	+	−
Reaction time trend	SR—Trend		−	+	−
Stroop^17^	ST	*Interference Control*			
N correct in congruent trials	ST—Cor Cong		+	−	−
N correct in incongruent trials	ST—Cor Incong		+	+	+
Reaction time congruent trials	ST—RT-M Cong		−	−	−
Reaction time incongruent trials	ST—RT-M Incong		−	−	−
Reaction time sd congruent trials	ST—RT-SD Cong		−	+	+
Reaction time sd incongruent trials	ST—RT-SD Incong		−	+	+
Go-Nogo^17^	GN	*Response Inhibition*			
N correct in go trials	GN—Cor Cong		+	+	+
N correct in no-go trials	GN—Cor Nogo		+	−	−
Reaction time go trials	GN—RT-M Go		−	−	−
Reaction time sd go trials	GN—RT-SD Go		−	+	+
Balloon Analogue Risk Task^21^	*BART*	*Risk Taking Behavior*			
N Pumps	BART—CorrPumps		+−	−	−
Change after burst occured	BART—∆Burst		+	+	−
N Bursts	BART—Burst		−	+	−
Total Points	BART—CorrTotal		+	+	−
NBack^17^	NB	*Interference Control*			
N correct n = 1 trials	NB—COR L1		+	+	+
N correct n = 2 trials	NB—COR L2		+	−	−
Reaction time n = 1 trials	NB—RT-M L1		−	−	−
Reaction time n = 2 trials	NB—RT-M L2		−	+	−
Reaction time sd n = 1 trials	NB—RT-SD L1		−	+	+
Reaction time sd n = 2 trials	NB—RT-SD L2		−	+	−
Tower of London^17^	TOL	*Problem Solving*			
Stimulus Duration (time needed)	TOL—StimDur		−	+	+
N correctly solved problems	TOL—COR		+	+	+
Kirby^17^	KIR	*Delay Discounting*			
Exponential discount rate value	KIR—ExpVal		−	−	−
Hyperbolic discount rate value	KIR—HypVal		−	+	−
Flanker^17^	FL	*Selective Attention*			
N correct in congruent trials	FL—Cor Cong		+	+	−
N correct in incongruent trials	FL—Cor Incong		+	−	−
Reaction time congruent trials	FL—RT-M Cong		−	−	−
Reaction time incongruent trials	FL—RT-M Incong		−	+	−
Reaction time sd congruent trials	FL—RT-SD Cong		−	+	+
Reaction time sd incongruent trials	FL—RT-SD Incong		−	+	−
Stop Signal^17^	SS	*Response Inhibition*			
N correct in all trials	SS—TotalCOR		+	−	−
N correct in go trials	SS—Cor Go		+	+	+
N correct in stop trials	SS—Cor Stop		+	−	−
Max delay value in high condition	SS—MaxDel High		+	−	−
Max delay value in low condition	SS—MaxDel Low		+	−	−
Reaction time in go trials	SS—RT-M Go		−	−	−
Reaction time sd in go trials	SS—RT-SD Go		−	+	+
Reversal Learning^22^	RL	*Cognitive Flexibility*			
N correct in all trials	RL—Correct		+	−	−
Change after probabilistic feedback	RL—PWF Change		+	−	−
N probabilistic errors	RL—ProbErr		−	+	−
N reversal errors	RL—RevErr		−	+	−
N total trials	RL—Trials		−	−	−
Ratio changes/PWF feedback	RL—PWF ChangeRatio		+	+	−
Judgement of Line Orientation^23^	LO	*Spatial Orientation*			
Reaction time	LO—TT-M		−	−	−
N correct	LO—COR		+	+	+
Distance to correct answer	LO—Dist		−	+	+
Information Sampling^17^	IS	*Decision Making*			
Reaction time decreased win	IS—RT DecWin		−	−	−
Reaction time fixed win	IS—RT FixWin		−	+	−
N clicks decreased win	IS—Clicks DecWin		+	+	+
N clicks fixed win	IS—Clicks FixWin		+	+	+
Neuropsychological Tests					
CERAD Plus^20^					
CERAD verbal fluency (animals)	CERAD ANIM (r)	*Verbal Fluency*	+		
CERAD Boston Naming Test	CERAD BNT (r)	*Lexical Retrieval*			
CERAD Mini Mental Status Examination	CERAD MMSE	*Lexical Retrieval*	+		
CERAD Word List	CERAD WL	*Memory*			
N correct word list 1	CERAD WL1		+		
N correct word list 2	CERAD WL 2		+		
N correct word list 3	CERAD WL 3		+		
Recall	CERAD WLREC		+		
Intrusions	CERAD INTRU		−		
Savings	CERAD WLSAV		+		
Recognition	CERAD WLRECog		+		
CERAD Figure	CERAD FIG	*Visuospacial ability/memory*			
Copying	CERAD FIGC		+		
Recognition	CERAD FIGR		+		
Savings	CERAD FIGSAV		+		
CERAD Trailmaking Test	CERAD TMT				
Time Trailmaking Test A	CERAD TMT-A	*Psychomotor speed*	−		
Time Trailmaking Test B	CERAD TMT-B	*Executive functioning*	−		
TMT-B/TMT-A	CERAD TMT-Ratio	*Executive functioning*	−		
CERAD S-Words	CERAD SW	*Phonematic Fluency*			
Wechsler Memory Scale Revised^24^	WMSR	*Memory*			
Digit Span forwards	WMSR DS-F		+		
Digit Span backwards	WMSR DS-B		+		
N correct total	WMSR DS-Total		+		
Brief Test of Attention^25^	BTA	*Shared Attention*			
Digit counting	BTA NUM		+		
Letter counting	BTA LET		+		
N correct total	BTA Total		+		
Visual Object and Space Perception^26^	VOSP	*Visual Perception*	+		
Wechsler Adult Intelligence Scale—Similarities^27^	WIE	Verbal Reasoning	+		
Questionnaires					
Non-motor Symptoms Questionnaire^28^	NMS-Quest	Non-motor Symptoms	−		
Parkinson’s Disease Sleep Scale 2^29^	PDSS		−		
Epworth Sleepiness Scale^30^	ESS Total	Daytime Somnolence	−		
Apathy Scale^31^	Apathy	Apathy	−		
Fatigue Severity Scale^32^	Fatigue (FSS)	Fatigue	−		
Beck’s Depression Inventory II^33^	Depression (BDI)	Depression	−		
Functional Activities Questionnaire^34^	FAQ Total	Functional independence	−		
EQ-5D^35^	EQ-5D	Health related quality of life			
Score	EQ-5D Index		+		
Visual Analogue Scale	EQ-5D VAS		+		
PDQ-8^36^	PDQ-8 Total		−		
10 Item Big Five Inventory^37^	BFI-10				
Extraversion	BFI Extraversion		+		
Openness	BFI Openness		+		
Agreeableness	BFI Agreeableness		+		
Conscientiousness	BFI Conscientiousness		+		
Emotional Stability	BFI Emotional Stability		+		
UPPS Impulsive Behavior Scale^38,39^	UPPS				
Urgency	UPPS Urgency		−		
Lack of Premeditation	UPPS Lack Premed		−		
Lack of Perseverance	UPPS Lack Persev		−		
Sensation Seeking	UPPS Sensat. Seek		−		
QUIP-RS^40^	QUIP-RS Total		−		
PGI^41^	PGI		+		
Disease Severity	PGI Severity		+		
Change in last 12 month	PGI Change		+		
Barratt Impulsiveness Scale 11^42^	BIS-11				
Attention	BIS-11 Attention		−		
Motor	BIS-11 Motor		−		
Nonplanning	BIS-11 Nonplanning		−		
Total	BIS-11 Total		−		
Obsessive–Compulsive Inventory -Revised^43^	OCI-R Total		−		
ICDRC^44^	ICDRC				
Gambling	ICDRC Gambling		−		
Sexuality	ICDRC Sexuality		−		
Shopping	ICDRC Shopping		−		
Eating	ICDRC Eating		−		
Repetitive Actions	ICDRC Repetitive		−		
Dopamine Dysregulation	ICDRC DDS		−		

*SD* standard deviation.

### Usability testing confirms the feasibility of the DiCo in an elderly population

A tablet-based cognitive assessment (DiCo) was developed by implementing 13 commonly used test paradigms that cover response inhibition, cognitive flexibility, attention, and working memory (Table 2). To make sure that the DiCo can be used efficiently in an elderly population, we placed particular emphasis on a user-friendly experience and on the clarity of test instructions. Training sessions were implemented to acquaint participants with the use of the tablet computer, in particular with the functionality of the touch input, and practice trials were incorporated into the assessments to provide participants with an opportunity to familiarize themselves with the testing procedures before the actual assessments. Comprehensive usability testing was conducted with a mixed-methods approach that incorporated quantitative measures and heuristic evaluations. DiCo results were not affected by PD motor symptoms, because there was no significant correlation between the MDS-UPDRS score and the simple reaction time (Supplementary Fig. 1), i.e., the DiCo feature that would be most likely to be affected by PD motor symptoms.

**Fig. 1:**
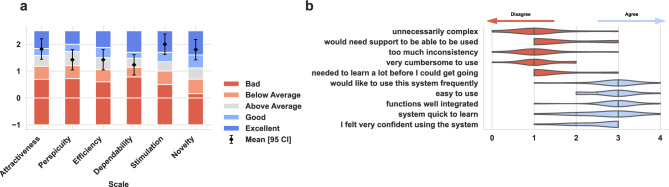
Usability testing reveals high overall satisfaction in established usability measures. (**a**) Overall UEQ ratings (**a**) for the DiCo (black diamond and whiskers) in the six established domains against the established benchmarks (colored bars). The measured scale means (± standard deviation) are set in relation to existing values from a benchmark data set. (**b**) SUS ratings from n = 15 PwPD participants of the usability testing. For each question, PwPD responded on a 5-item Likert scale (strongly disagree, disagree, neutral, agree, strongly agree).

Quantitative measures were obtained through the System Usability Scale (SUS)^45^ and the User Experience Questionnaire (UEQ)^46^. The SUS is a widely used questionnaire that assesses the overall usability of a system, generating a numeric score that reflects user satisfaction. SUS ratings revealed a high overall satisfaction with the system, while some participants expressed the need for support and some time to get familiar with the use (Fig. 1c). The UEQ, on the other hand, provides an evaluation of the user experience across multiple dimensions, including attractiveness, perspicuity, efficiency, and stimulation. The cognitive testing battery achieved high scores in the UEQ domains of attractiveness (1.88 ± 0.81), stimulation (2.00 ± 0.84), and novelty (1.80 ± 0.98), indicative of a high “hedonic quality” (Fig. 1b). The rating for the pragmatic quality was above average (indicated by perspicuity, efficiency and dependability, mean 1.36).

### DiCo captures performance-based dimensions that are stable across analytic approaches

Completing the entire assessment took 67 min on average, the interquartile range was 59–71 min. From each test, several different features were extracted, including mean and standard deviation of the reaction time, percentage of correct answers and others. In total, 54 features were extracted from the 13 tests (Table 2). Measures of central tendencies and split-half reliability for each DiCo task and features are listed in Supplementary Table 1.

To determine whether the extracted DiCo features reflect cognitive domains, task-specific variance, or broader performance dimensions, we conducted three complementary analyses: hierarchical cluster analysis, network modeling and exploratory factor analysis (EFA).

**Fig. 2:**
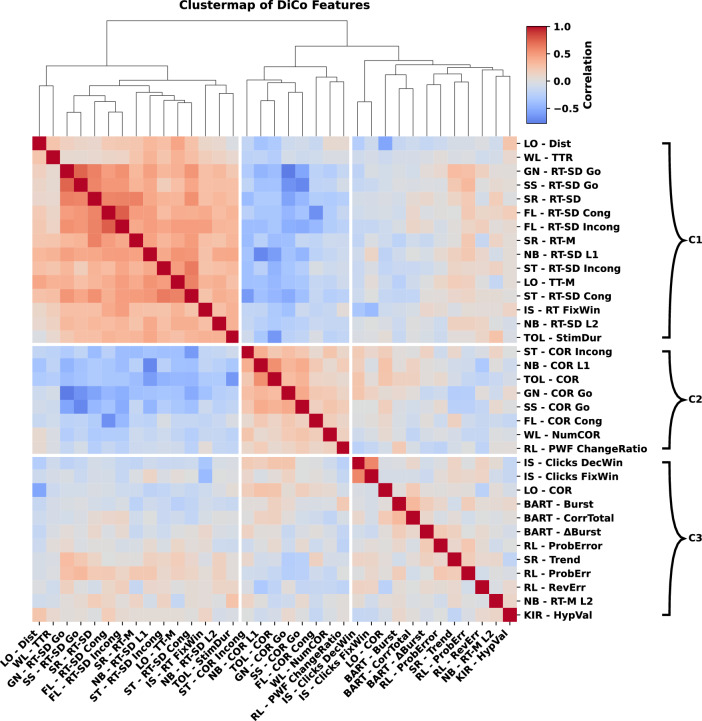
Cluster map of DiCo feature correlations revealing three clusters. C1 includes reaction time and timing-related measures. C2 comprises accuracy-based variables that reflect actual task performance in terms of correctness. C3 represents a residual group, primarily consisting of features from impulsivity-related tasks. Supplementary Fig. 2 displays the same analysis with measures in cluster 1 inverted. Abbreviations used in this figure are defined in Table 2.

The hierarchical cluster map (Fig. 2) was based on pairwise feature correlations and revealed three interpretable clusters of features: time-based measures such as response time (C1), accuracy-related measures (C2), and a residual cluster mainly comprised of features from BART, Information Sampling and Reversal Learning (C3). Correlation Coefficients were stronger within C1 and within C2 than within C3, indicating less internal coherence within the third cluster. Because good performance is associated with short response times and high accuracy, we inverted the time-based measures in C1 to align higher values with better performance. After this transformation, the cluster structure changed and yielded two main clusters (Supplementary Fig. 2): one combining response time and accuracy measures across all cognitive tests, and a residual cluster similar to C3. Taken together, the hierarchical cluster map revealed a close association between features for good performance across different tests.

**Fig. 3:**
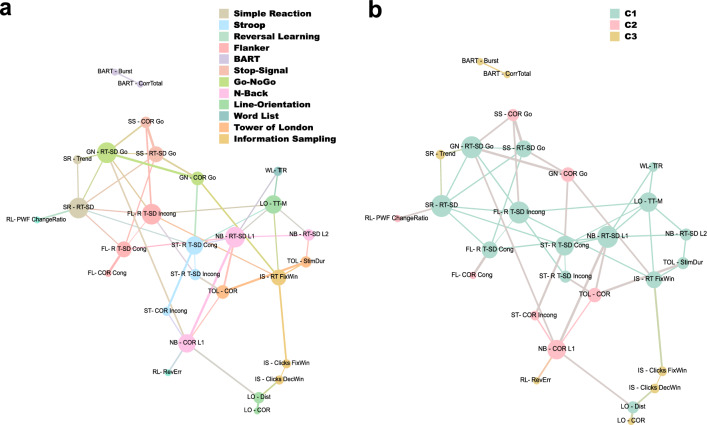
Conditional dependencies within the same cognitive test and across performance-based features. A graphical lasso was used to estimate a sparse graph of undirected relationships between all digital variables (**a** and **b**). Variables were colored according to the parent test (**a**) or according to their association with the clusters from Fig. 2 (**b**) Edges in the graph reflect absolute conditional dependency greater than 0.10. The size of the nodes increases with the number of connections; the thickness of the edges increases with stronger absolute conditional dependency.

To confirm this finding with a different method, we calculated the sparse inverse covariance matrix (precision matrix) using the graphical Lasso algorithm. The precision matrix identifies conditional dependencies between individual variables while controlling for the influence of all others. Color-coding the resulting network structure based on tests (Fig. 3a) showed strong within-test connectivity and sparse connections between tests: Whereas 66.7% (n = 14 of 21) of connections within the same test were realized, only 10.1% (n = 36 of 357) of connections across tests were realized.

To align this clustering of nodes from the same test with the strong correlations between tests observed above, we color-coded the nodes of the network graph (Fig. 3b**)** according to the three variable clusters derived from the hierarchical cluster map (Fig. 2). The cluster assignments aligned remarkably well with the structure of the network that was derived from the precision matrix. Particularly, time-based features (C1) formed a densely interconnected core, whereas impulsivity-related measures (C3) appeared largely isolated from the rest of the network. This convergence confirms the structural validity of our findings across analytic approaches.

**Fig. 4:**
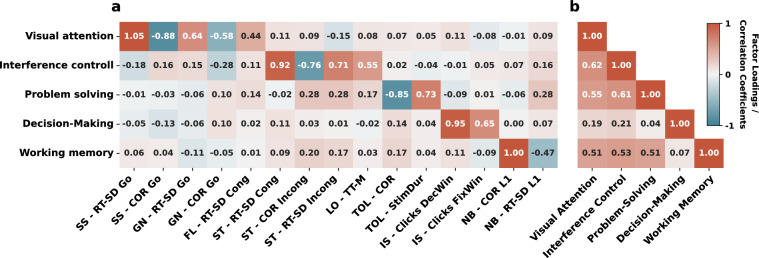
Exploratory factor analysis reveals four intercorrelated latent factors. (**a**) Five extracted factors and factor loadings of DiCo variables. (**b**) Correlations between the five extracted factors. SS = StopSignal, GN = Go-Nogo, FL = Flanker, ST = Stroop, LO = Judgement of Line Orientation, TOL = Tower of London, IS = Information Sampling, NB = NBack-Task; Further abbreviations used in this figure are defined in Table 2.

Hierarchical clustering and network analysis describe the observed data structure without modeling underlying sources of shared variance. Such latent dimensions could be analogous to the concept of cognitive domains and allow a theoretically meaningful interpretation of the observed covariance structure. To identify latent dimensions, we conducted an exploratory factor analysis (EFA). After excluding features with low sampling adequacy (Kaiser–Meyer–Olkin Test, KMO < 0.50), low communalities (< 0.40) and eigenvalues < 1.0 ^47^, the EFA yielded five factors (Fig. 4a). These factors explained 64.6% of the total variance, exceeding the commonly used threshold of 60% in social sciences^48^. The first factor consisted of features from StopSignal, Go-Nogo and the Flanker task, specifically those derived from the “Go” conditions that did not require inhibition or discrimination. Since those trials primarily capture aspects of attention, we chose to name this factor “visual attention”. The remaining four factors mostly consisted of variables from the same test and were thus named after the cognitive domain these tests have been associated with: Stroop—*Interference control*, Tower of London—*Problem solving*, Information Sampling—*Decision-Making*, and N-Back—*Working Memory*. Because these factors were largely task-defined, they may partly reflect task-specific variance in addition to domain-relevant cognitive processes. We refer to them as DiCo factors from here on.

We then tested for correlations between DiCo factors. The correlation of *Decision Making* with the other factors was small (Fig. 4b), but the remaining four factors were moderately to strongly interrelated with correlation coefficients ranging from r = 0.51 to 0.62. This high degree of interrelatedness suggests a considerable degree of shared variance across cognitive domains. The shared variance across the four factors is consistent with the finding from hierarchical clustering (Supplementary Fig. 1) and network analysis (Fig. 3b).

### Construct validity of DiCo factors

**Fig. 5:**
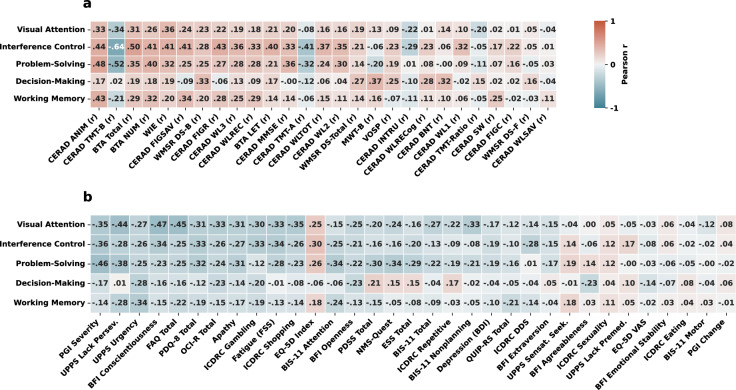
Construct validity of DiCo factors. Depicted are Pearson correlations between DiCo-derived factors and external neuropsychological measures (**a**) as well as self-report questionnaire data (**b**). Neuropsychological data were available for n = 50 and questionnaire data for n = 77 participants. Abbreviations used in this figure are defined in Table 2.

To evaluate the construct validity of the identified DiCo factors, we calculated Pearson correlation coefficients with summary scores obtained from neuropsychological tests (NPT) and validated questionnaires (Q) (Fig. 5; Supplementary Tables 2 and 3). We only observed moderate associations; the strongest correlation coefficients with a DiCo-derived factor ranged between r = 0.30 and r = 0.64. Due to the coding schemes of the individual variables (Table 2), we expected DiCo factor scores to correlate positively with neuropsychological measures for which higher scores indicate better performance and negatively with timed neuropsychological measures such as TMT-A and TMT-B, for which longer completion times indicate poorer performance. For questionnaire measures of symptoms or impairment, we expected negative correlations with DiCo factor scores, whereas measures of quality of life or preserved function were expected to correlate positively.

The DiCo factor *Visual Attention* was moderately associated with the following variables obtained by NPT (Fig. 5a): finding similarities (WIE; r = 0.36), executive functions (CERAD TMT-B, r = − 0.34) and semantic verbal fluency (CERAD ANIM, r = 0.33). *Interference Control* was most strongly correlated with executive functioning (CERAD TMT-B, r = − 0.64). *Problem-Solving* was related to semantic verbal fluency (CERAD ANIM, r = 0.48) and executive functioning (CERAD TMT-B, r =  − 0.52). *Working Memory* was most strongly related to semantic verbal fluency (CERAD ANIM, r = 0.43). *Decision Making* showed only weak associations with the NPT variables, except for a moderate correlation with vocabulary knowledge (MWT-B, r = 0.37). This latter association may reflect a response style where participants prematurely accepted options as valid without careful evaluation potentially mirroring increased reflection impulsivity.

For the questionnaire data (Fig. 5b), the DiCo factor *Visual Attention* showed moderate negative relationships with conscientiousness (BFI Conscientiousness, r = − 0.47), functional abilities in everyday life (FAQ total, r = − 0.45) and lack of perseverance (UPPS Lack Persev., r = − 0.44). *Interference Control* was weakly to moderately related to PwPD’ global impression of condition (PGI Severity, r = − 0.36), fatigue (Fatigue FSS, r = − 0.34) and gambling (ICDRD Gambling, r = − 0.33). *Problem-Solving* was most strongly related to the participants subjective global impression of condition (PGI Severity, r = − 0.46) and moderately related to lack of perseverance (UPPS Lack Persev., r = − 0.38). *Working Memory* overall also showed weak correlations to Q-Data variables with urgency (UPPS Urgency, r = − 0.34) and lack of perseverance (UPPS Lack Persev., r = − 0.28) being the most prominent. *Decision-Making* again showed only weak correlations to questionnaire variables with urgency (UPPS Urgency, r = − 0.28) being the strongest.

In addition to the factor-level correlations, we calculated Pearson correlation coefficients between the raw DiCo features and paper-and-pencil neuropsychological tests as well as questionnaires to assess convergent patterns at the feature level (Supplementary Fig. 3).

### Profile analysis identifies three clusters with different levels of performance and one cluster with a distinct profile in the factor *Decision-Making*

**Fig. 6:**
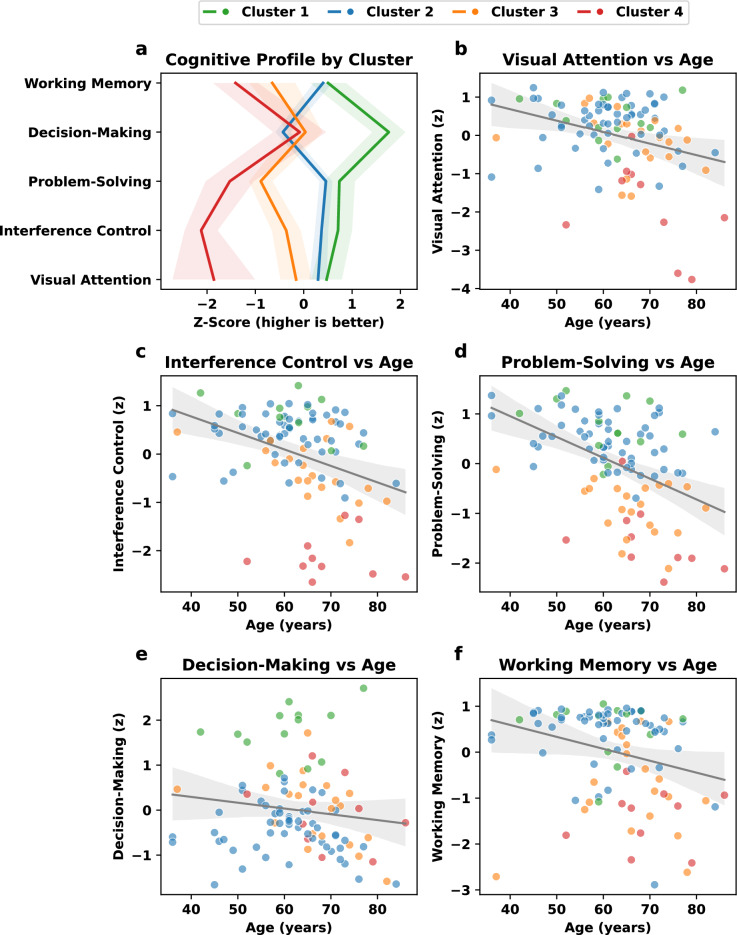
Latent profile analysis on DiCo factor scores revealed four clusters. (**a**) Participants were clustered by their factor scores using Latent Profile Analysis. Clusters 1 (n = 13), 3 (n = 21) and 4 (n = 10) differed consistently across all factors, whereas cluster 2 (n = 53) performed similarly to cluster 1 in four of the five factors but significantly worse in Decision-Making. (**b**–**f**) Associations between each factor and age with data points color-coded according to the cluster membership. All factors except Decision-Making showed significant age-related decline.

We then used this insight to identify potential differences in cognitive profiles between PwPD, by conducting a Latent Profile Analysis using DiCo factor scores. Based on the fit-indices obtained for solutions with 1 to 5 clusters (Supplementary Table 4), we selected a four-cluster solution (Fig. 6a). PwPD in clusters 1, 3 and 4 differed quantitatively with homogenous differences across all DiCo factors. Cluster 2 performed similarly to cluster 1 in four of the five domains but significantly worse in *Decision Making*—ranking lowest among all clusters. We refer to these four clusters of PwPD as DiCo clusters from here on. Clinical data of PwPD for the three clusters is summarized in Table 3.

We hypothesized that DiCo clusters 1, 3, 4 represent stages of gradual decline. The homogenous differences between these clusters suggest that decline in PD progresses rather homogenously across DiCo factors. In order to test whether this gradual decline is driven by age or by PD, we calculated linear regression models for each DiCo factor score with age as predictor (Fig. 6b–f). Four factors revealed significant negative slopes with age, ranging from − 0.026 to − 0.042, suggesting that four DiCo factors decline with increasing age. Decline was particularly strong for the DiCo factor *Problem-Solving*. Color coding PwPD based on DiCo clusters suggests that performance differences are not primarily driven by age. For instance, DiCo cluster 1 consistently performed above the age-predicted trend lines, whereas DiCo cluster 4 scored below cluster 1 for all domains.

In contrast to most DiCo factors, *Decision-Making* appeared independent of age in this cohort (Fig. 6e). This distinct property of *Decision-Making* is consistent with its poor correlation with the other DiCo factors (Fig. 4b), NPT data (Fig. 5a) and questionnaires (Fig. 5b). It is also consistent with fact that the DiCo factor *Decision-Making* separates DiCo clusters 1 and 2 (Fig. 6a).

### Exploratory longitudinal analyses suggest changes primarily affect composite motor score, working memory, problem-solving and interference control

**Fig. 7:**
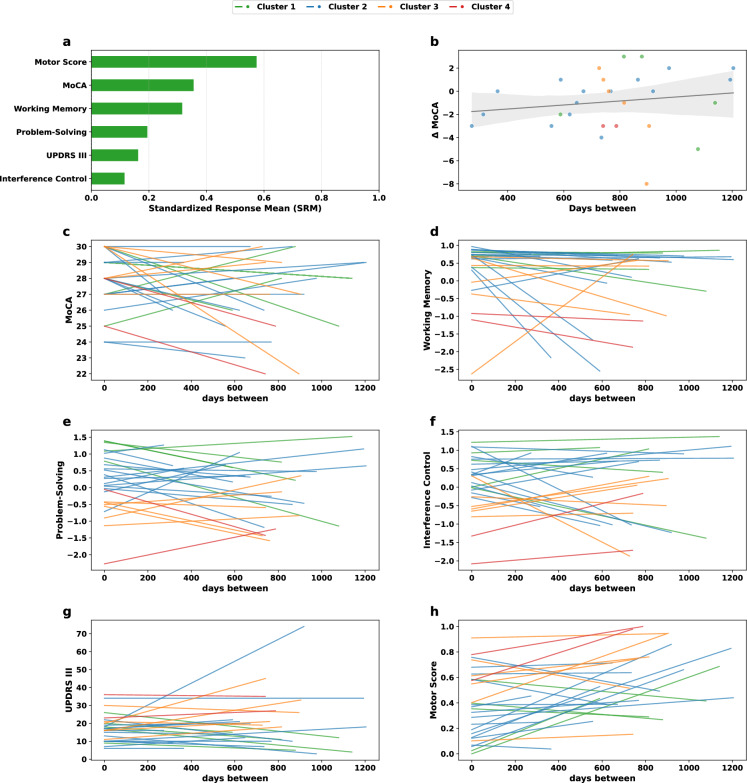
Longitudinal analyses of cognitive and motor outcomes. The UMAP-derived motor score yielded the highest SRM with MoCA and Working Memory being almost equally sensitive to change (**a**). MoCA scores showed slight improvements with increasing time between measurements (**b**). Individual trajectories for MoCA as well as the three DiCo-derived factors Working Memory, Problem-Solving and Interference Control (**c**–**f**). While UPDRS III remained relatively stable from baseline to follow-up (**g**), the UMAP-derived motor score showed progression in almost every participant (**h**).

Longitudinal assessments were available for a small convenience subset of our cohort and were therefore analyzed exploratorily. The mean delay between the two assessments was 25 months. To compare cognitive changes across domains and with respect to motor changes, we computed Standardized Response Means (SRMs), defined as the mean change divided by the standard deviation of the change scores (Fig. 7a). Next to SRMs for each DiCo factor, we used the MoCA score as a commonly used cognitive assessment and the motor score UPDRS III. Because the UPDRS III can be affected by dopaminergic medication, we determined, in addition, a compound motor score using Uniform Manifold Approximation and Projection (UMAP).

The effect size of change over time was moderate for the UMAP-derived motor score (SRM = 0.57), small to moderate for MoCA (SRM = − 0.36) and *Working Memory (SRM* = *− 0.32)*, and only very small for *Problem-Solving* (SRM = − 0.19), UPDRS III (SRM = 0.16) and *Interference Control* (SRM = − 0.11). Accordingly, individual trajectories showed stronger changes for the compound motor score (Fig. 7h) than for MoCA (Fig. 7c) and *Working Memory* (Fig. 7d). Paired t-tests showed significant differences between baseline and follow-up values for the compound motor score (t = − 2.857, p = 0.008), but not for UPDRS III (t = − 0.844, *p* = 0.405) or MoCA (t = 1.424, *p* = 0.165).

The UMAP-derived motor score showed the strongest change over time but only a weak correlation with age (r = 0.27; Supplementary Fig. 4). This pattern may indicate that longitudinal trajectories in this subset were more closely related to disease- or treatment-related progression than to age alone. Conversely, *Problem Solving* showed the most pronounced age-dependent differences between PwPD (Fig. 6d) but relatively small longitudinal changes within one PwPD (Fig. 7e). This discrepancy is consistent with the hypothesis that aging and PD differentially affect the cognitive profile.

### Associations of digital cognitive assessments with MoCA and FAB

To investigate the relationship of features obtained from the DiCo with overall cognition and executive function, we trained two separate Random Forest Regressors to predict the scores of the Montreal Cognitive Assessment (MoCA, Model 1) and the Frontal Assessment Battery (FAB, Model 2). DiCo factor scores, sociodemographic and clinical variables were used as predictor variables. Hyperparameter tuning was achieved via grid search and fivefold cross-validation. Model performance and feature importances were evaluated across multiple random seeds using repeated cross-validation (5 folds, 2 repeats), resulting in a robust estimation across 150 model runs. Mean absolute Error (MAE) and Root Mean Squared Error (RMSE) were computed to evaluate prediction performance. To identify the most relevant predictors for MoCA and FAB, our main interest was in the averaged feature importances and their standard deviation across all folds and random seeds.

**Fig. 8:**
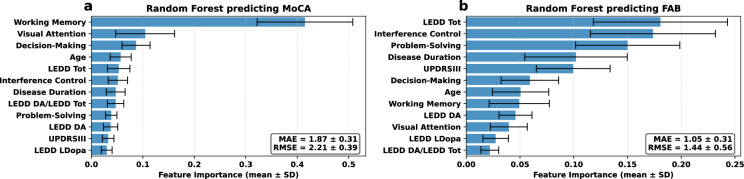
Associations between DiCo factors and cognitive screening tests. We trained Random Forest models to predict MoCA (**a**) and FAB (**b**) scores using DiCo factor scores alongside sociodemographic and clinical variables. Prediction accuracy was evaluated across 150 repeated cross-validation runs. For MoCA, Working Memory and Visual Attention were the most important predictors. For FAB, feature importance was more evenly distributed, with Interference Control and total LEDD being most influential.

For Model 1, grid search revealed the following best hyperparameters: n_estimators = 50, min_samples_split = 2,min_sample_leaf = 4, max_depth = 10. Model 1 predicted the MoCA scores with a MAE of 1.87 (SD = 0.31) and a RMSE of 2.21 (SD = 0.39). These values indicate that, on average, the model’s predictions are reasonably close to the actual scores. *Working Memory* was, by far, the most important DiCo factor for predicting the MoCA score (Fig. 8a).

For model 2, grid search yielded n_estimators = 100, min_samples_split = 5, min_samples_leaf = 2 and max_depth = 5 as optimal hyperparameters. Model 2 predicted the FAB scores (Fig. 8b) with a MAE of 1.05 (SD = 0.31) and a RMSE of 1.58 (SD = 0.56). In contrast to Model 1, no single feature stood out for predicting the FAB. Total levodopa equivalent dose and *Interference Control* were almost equally important for predicting the FAB, followed by *Problem-Solving* and *Disease Duration*. The more evenly distributed feature importance could be explained, for instance, by co-linearity among predictors.

**Fig. 9:**
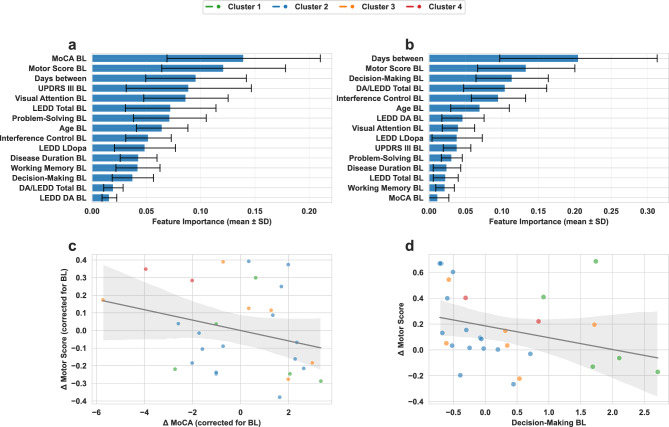
Predictors of cognitive and motor changes. Relative feature importances from Random Forest regressors predicting changes in MoCA (**a**) and UMAP-derived Motor Score (**b**). For the prediction of motor decline, baseline (BL) DiCo-derived Decision-Making was among the most important predictors, following time between assessments and baseline motor performance. Baseline-corrected changes in MoCA and Motor Score (**c**) were only weakly negatively correlated, suggesting that cognitive and motor progression may occur independently. Additionally, lower Decision-Making performance at baseline (**d**) were associated with greater motor deterioration.

We then trained a second set of random forest regressors to predict the change in MoCA and the change in the compound motor score over time. To predict the change in MoCA, the MoCA and compound motor scores at baseline were the most important features, followed by the time between the two assessments and UPDRS III at baseline (Fig. 9a). To predict the change in Motor Score, time between the assessments, motor score at baseline and *Decision Making* were the most important features (Fig. 9b). Interestingly, baseline *Decision-Making* was associated negatively with change in Motor Score (r = − 0.31, *p* = 0.11; Fig. 9d), indicating that higher reflection impulsivity may predict stronger motor decline over time. Given the small number of participants (n = 28) in the longitudinal cohort, these results need to be interpreted with caution.

After controlling for baseline values, the residualized MoCA and motor change scores only exhibited a weak correlation (r = − 0.28, p = 0.15; Fig. 9c), suggesting that the progression of motor and cognitive performance over time may develop independently from one another.

## Discussion

In this study, we developed and validated an open-source tablet-based cognitive assessment battery for PwPD. 54 features were obtained in 13 test paradigms. Three structural analyses were conducted, which converged on the notion that cognitive performance of PwPD without manifest cognitive impairment, as captured by the DiCo, is characterized by strong intercorrelations across domains. Specifically, the main difference between clusters C1 and C2 obtained by correlation-based clustering (Fig. 2) consisted in the direction of changes, and inverting the time-based measures resulted in one cluster combining both response time and accuracy measures across cognitive tests, and another residual cluster similar to the original C3 (Supplementary Fig. 2). In the network analysis, features clustered within the same test (Fig. 3a) and along the clusters C1 and C2 (Fig. 3b), but not along other domains. The EFA revealed five latent factors, four of which were strongly interrelated (Fig. 4b). These factors also correlated moderately with neuropsychological test results (Fig. 5a) and questionnaire data (Fig. 5b). Clustering of individual PwPD showed homogenous differences between 3 of 4 clusters (Fig. 6a), which can be classified as severity-based using the nomenclature by Pourzinal et al.^49^. These results are in line with analyses of cognitive test scores obtained in 698 PwPD from the DEMPARK and LANDSCAPE cohorts^50^,^51^.

This rather uni-dimensional structure of test results consistent with a mutualistic, network-like structure of cognitive abilities as suggested by recent theoretical developments in cognitive science^52^, and with hierarchical neuropsychological models of cognition that include a dominant higher-order factor (e.g. “g-factor”) next to strictly separable domains such as attention, working memory, executive functions, language, memory and visuospatial abilities. The severity-based differences are found across all tests of the DiCo and larger than the effect of age (Fig. 6b–f). This comparison provides an estimate of the magnitude of cognitive differences in PwPD, even before the onset of overt cognitive impairment.

The severity-based difference is not only larger than the effect of age, it also affects different cognitive functions: Whereas the DiCo factor *Problem Solving* was most strongly associated with age (Fig. 6d), the DiCo factor *Working Memory* changed most strongly between baseline and follow-up (Fig. 7a).

The DiCo factors correlated moderately with established paper–pencil neuropsychological tests (Fig. 5a). Given the substantial methodological differences, this correlation strength is not unexpected. Previous studies comparing paper–pencil versions of a neuropsychological test with its exact digital implementation found correlations of 0.63 for TMT-A, 0.77 for TMT-B, and 0.68 for the Color-Word Interference Test^53^. Assessing the relationship between the processing speed subtests *Coding* and *Symbol Search* from the Wechsler Intelligence Scale for Children, Ferriola^54^ found correlations of 0.67 and 0.61 respectively. All these were comparisons of exact digital representations with their paper–pencil versions. We therefore interpret these as the upper bound of convergence validity that can be expected across testing modalities. In our case, digital assessments were compared to neuropsychological tests that were not exact implementations of one another. This can explain the lower correlation coefficients we observed. This interpretation is in line with findings from Mielke et al.^55^ who found absolute correlations between digital tests from the CogState digital test battery and paper–pencil tests between 0.46 and 0.53. Finally, the DiCo factor *Visual Attention* represents a composite of features from three different tests.

The relatively weak associations between DiCo-derived features and questionnaires are in line with previous work reporting limited correspondence between task-based and self-report measures of self-regulation, as demonstrated by Eisenberg et al.^56^, who found a near-complete separation of task and survey variables in a multivariate behavioral space.

The associations we did observe between DiCo factors and paper–pencil tests are theoretically plausible. For example, *Interference Control* and the TMT-B have both been linked to executive functioning^57,58^. Similarly, *Problem-Solving* and verbal fluency have both been linked to microstructural white matter changes in newly diagnosed PwPD, suggesting a shared neuroanatomical basis^59^. For questionnaires, most associations to DiCo factors were even more moderate (Fig. 5b). Interestingly, the correlation between *Problem-Solving* and participants’ subjective impression of disease severity was rather strong, which underlines previous findings that cognitive symptoms play an important role in PwPDs’ quality of life^60–62^.

The DiCo factor *Decision-Making* consistently emerged as rather independent, being less correlated with the other factors (Fig. 4b), less correlated with neuropsychological test results and questionnaire data (Fig. 5). *Decision-Making* did not decline with age (Fig. 6e) and separated the two largest clusters of PwPD (Fig. 6a): cluster 2 showed similar performance as cluster 1 in most DiCo factors but was significantly impaired in *Decision-Making*. The DiCo factor *Decision-Making* contributed little to the prediction of MoCA and FAB (Fig. 7).

*Decision-Making* was mainly driven by features of the information sampling test (Fig. 4a). The information sampling test responds to reflection impulsivity, i.e., the tendency to make rapid decisions without sufficient information gathering or consideration of alternatives. Dopamine agonists increase reflection impulsivity^63^ and all dopaminergic treatment can cause impulse-control disorders^64–66^. This raises the possibility that the *Decision-Making* feature results not from aging or PD, but from dopaminergic medication. Interestingly, *Decision-Making* was associated with changes in the composite motor score (Fig. 10b), which includes medication information next to the UPDRS III (Supplementary Fig. 5). In addition, we observed a trend where a higher proportion of dopamine agonists in the medication profile was associated with poorer performance on the information sampling task—particularly among participants who performed well on other cognitive tests (cluster 2 vs. cluster 1, Table 3).

**Table 3:** Comparison of clinical variables between clusters

Feature	Cluster 1 (N = 13)	Cluster 2 (N = 53)	Cluster 3 (N = 21)	Cluster 4 (N = 10)	Kruskal–Wallis	Mann–Whitney U	Mann–Whitney U (Holm-corrected)
Age (years)	60.7 (9.0)	60.7 (10.1)	66.2 (9.6)	69.5 (9.4)	*p* = 0.013	1,2 < 3,4	n.s
Disease duration (years)	6.7 (3.3)	5.8 (4.6)	6.7 (5.3)	8.1 (5.1)	*p* = 0.403	n.s	n.s
Age at onset (years)	54 (8.9)	54.6 (10.1)	59.5 (10.6)	61.4 (12.33)	*p* = 0.039	2 < 3	n.s
Sex (% male)	61.5	58.5	52.3	50	χ^2^ = 0.53, *p* = 0.91		
LEDD Total (mg)	685.6 (283.5)	627.3 (355.4)	721.8 (345.6)	789.6 (298.5)	*p* = 0.272	n.s	n.s
LEDD LDopa (mg)	420.5 (168.8)	355.0 (177.0)	496.0 (223.3)	603.6 (105.5)	*p* < 0.001	2 < 3; 1,2 < 4	2 < 4
LEDD DA (mg)	204.4 (106.7)	253.3 (109.3)	206.4 (158.1)	338.5 (300.4)	*p* = 0.159	n.s	n.s
DA/LEDD Total (%)	33.6 (18.0)	44.6 (22.4)	25.6 (17.6)	35.2 (32.3)	*p* = 0.012	2 > 3	2 > 3
Vascular risk present (%)	23.1	37.7	28.6	50	*p* = 0.449	n.s	n.s
UPDRS III	18.8 (9.1)	17.4 (8.7)	21.3 (7.6)	25.9 (10.8)	*p* = 0.047	2 < 4	n.s
MoCA	27.4 (1.8)	26.9 (2.3)	25.6 (3.1)	23.6 (3.5)	*p* = 0.011	1,2 > 4	2 > 4
FAB	16.8 (1.4)	17.0 (1.3)	16.6 (1.5)	13.5 (3.4)	*p* = 0.004	1 ,2,3 > 4	1,2,3 > 4
SCD (%)	38.5	47.2	71.4	80.0	*p* = 0.058	n.s	n.s

Kruskal–Wallis test was used to assess group differences across the four clusters, as the data were not normally distributed and group sizes were unequal. Given the small sample sizes in some clusters, we chose pairwise Mann–Whitney U tests for post-hoc comparisons, as this approach is less conservative and more robust than Dunn’s test in small samples. For each comparison, both the uncorrected and Holm-corrected differences are reported. n.s.—not significant, LEDD—levodopa equivalent daily dose; DA—dopamine agonist; MoCA—montreal cognitive assessment, FAB—frontal assessment battery, SCD—subjective cognitive decline.

In this study, cluster assignment based on DiCo-derived factor scores revealed four subgroups of PwPD with distinct performance profiles (Fig. 6a). These four clusters were poorly explained by age, sex, disease duration, age at disease onset, presence of cardiovascular risk factors, and motor impairment (Table 3). Also, the classical tests MoCA and FAB did not differ significantly between clusters 1, 2 and 3 (Table 3). The digital assessment implemented in this study can therefore identify differences that are not observed with these measurements. This might result from the fact that the DiCo measures slightly different properties than paper–pencil tests (Fig. 5). In addition, digital assessments might provide finer granularity.

Although DiCo showed good usability, the full assessment required approximately one hour and is therefore better suited for comprehensive research phenotyping than for routine outpatient screening, where brief tools such as MoCA and FAB remain more appropriate. Participant burden should also be considered for repeated longitudinal monitoring, particularly in older PwPD or those with fatigue, motor symptoms, or cognitive impairment. Because DiCo is modular, the present findings may inform reduced task sets for specific research questions or selected clinical applications. In this context, the uni-dimensional structure of the DiCo results implies that the DiCo could be simplified to reduced patient burden. In the subset of PwPD where longitudinal data was available, the DiCo factor *Working Memory* exhibited the largest standardized response mean (SRM) between baseline and follow-up, comparable to the MoCA. Results from the N-Back task loaded mainly on *Working Memory* (Fig. 4a). Consequently, the N-Back task may be a candidate for future reduced task sets assessing general cognitive performance in longitudinal research in PwPD, analogous to MoCA, but such shortened versions require prospective validation.

The very low correlation between residual changes in MoCA and UMAP-based composite motor score (Fig. 10c) underscores the importance of examining cognitive and motor progression as partly independent trajectories in PwPD.

Longitudinal effects were not observed when using the UPDRS III instead of the composite motor score. In fact, we did not observe a significant difference in UPDRS III scores between baseline and follow-up (Fig. 8d). Similarly, Holden et al. found UPDRS III to increase 4 points per year in unmedicated PwPD whereas PwPD on medication progressed only 1.2 points per year.^67^ Dopaminergic medication can therefore mask motor progression and reduce the sensitivity of UPDRS III to subtle longitudinal changes. The significant differences observed between baseline and follow-up in the composite score suggest that integrating motor ratings and medication burden may increase sensitivity to longitudinal motor changes. However, the UMAP-derived composite score is exploratory and should not be interpreted as a validated clinical motor endpoint. Because UMAP is sample-dependent and does not provide explicit feature weights, the sensitivity and interpretability of this composite score require validation in larger cohorts with standardized medication-state assessments.

Our sample of 97 PwPD primarily included participants in the transition phase of PD without overt cognitive impairment. As for other cohorts in early PD^68^, this potentially limited the detection of dementia-related patterns, but allowed for an analysis of subtle cognitive differences that may already be present in early stages of the disease. The sample size should be considered when interpreting the factor analysis, latent profile analysis, and prediction models. Although the cohort size is comparable to previous multidimensional cognitive profiling studies^69,70^ in Parkinson’s disease, the exact factor solution, profile structure, and feature-importance rankings require replication in independent cohorts. For longitudinal assessments in particular, the number of PwPD included in the study was small and analyses need to be interpreted with caution, especially because the follow-up sample was a convenience subset that appeared cognitively healthier at baseline, limiting the generalizability of longitudinal findings to the full baseline cohort. Moreover, test–retest reliability was not assessed across the full sample, and longitudinal changes may partly reflect practice effects. Because participants were recruited in an outpatient clinic, PwPD with impaired mobility were underrepresented in our study, limiting the generalizability of the feasibility assessments to PwPD with advanced disease, more severe cognitive impairment, impaired mobility, or greater fatigue. Moreover, the cohort is quite homogenous with respect to ethnic disparities. Multicenter validation studies are therefore required. We hope that the availability of the app on github along with the test implementations will facilitate such confirmation studies. Furthermore, analyses focused on the EFA-derived factors. As a consequence, features from the Simple Reaction, Kirby Delay Discounting, BART, and Reversal Learning tasks were not included in these analyses and remain to be evaluated in future work.

In summary, this study describes a comprehensive digital cognitive assessment that has been extensively usability-tested and can be readily applied for research on cognitive impairment in PwPD. Our analyses suggest, however, that cognitive impairment may change predominantly along a shared severity-related dimension in PD, so a simplified assessment could be sufficient for longitudinal studies. The prognostic value of these assessments needs to be confirmed in longitudinal studies with a higher number of participants.

## Methods

### Study population and design

The study was approved by the institutional review board of the Technische Universität Dresden (BO-EK-494112020). All investigations were performed in accordance with relevant guidelines and regulations, including the Declaration of Helsinki. Written informed consent was obtained from all participants prior to any investigation. Participants were recruited at the Department of Neurology of the Dresden University Hospital. In this observational exploratory study, a sample size of 100 cases was targeted. To ensure a diverse study population, the inclusion criteria for our study were deliberately broad, encompassing individuals aged 18 and above who met the diagnostic criteria for PD as stipulated by the Movement Disorder Society^71^. Individuals with severe cognitive or psychiatric disorders that could impede the successful completion of the cognitive test battery were excluded. Clinical and demographic data were collected, including motor and cognitive status (Hoehn & Yahr stage, UPDRS part III, Montreal cognitive assessment) as well as the presence of disease-related complications.

### Selection and implementation of tests

The selection of digital cognitive tests for our study was informed by a literature review centered on cognition in PD. We systematically examined research findings and consensus-based recommendations and identified specific cognitive domains implicated in PD pathology, with a particular focus on response inhibition, cognitive flexibility, attention, and working memory^72^. A data-driven identification of cognitive subtypes identified two types of clustering models: severity-based and domain-based. The majority of severity-based models revealed three distinct clusters of PwPD. The domain-based models identified two to six clusters with mnestic, visuospatial, executive and attentional impairment being the most common subtypes^49^.

The 13 selected tests were implemented using the open-source JavaScript framework *jsPsych,* optimized for touch-input and deployed via an *Experiment Factory* Container. To ensure a distraction-free and uniform testing environment, an iOS client application was developed. The app as well as the test implementations are available on github:. All participants conducted the test on an 8th-generation Apple iPad at maximum display brightness.

The app was designed to have participants undergo a brief instructional session to familiarize them with the touch controls prior to the actual experiments. During the instructional session, participants were required to accurately tap a circular area displayed on the screen. Each successful tap was met with immediate feedback, designed to reinforce correct interactions and improve proficiency of the touch control system. This preliminary exercise ensured that all PwPD were comfortable and adept at using the touch interface, allowing for a consistent starting point in the subsequent experimental tasks.

All participants conducted the tests in a strategically designed order optimized for participant engagement and balanced for cognitive strain. Demanding tests that required high levels of concentration and attention were interleaved by shorter, less strenuous tasks that in the usability testing were perceived as more enjoyable or attractive. This approach was intended to mitigate fatigue and maintain participant motivation throughout the testing session. Additionally, simpler tasks where intense attention and quick reaction times were not the primary focus (e.g. the Information Sampling Task) were included in the second half of the test series.

### Usability testing

Usability testing employed a mixed-methods approach that incorporated heuristic evaluations with experts and quantitative, as well as qualitative measures, in PwPD. The Systems Usability Scale (SUS) provides a subjective assessment of usability by the participant and involves a set of ten Likert scale questions^45^. Participants provide ratings on a scale from 1 to 5 (strongly disagree to strongly agree) for odd-numbered questions and from 5 to 1 (strongly agree to strongly disagree) for even-numbered questions. The SUS-score is calculated by subtracting 1 from the user’s response for odd-numbered questions and subtracting the user’s response from 5 for even-numbered questions, summing these adjusted scores, and then multiplying the total by 2.5 to yield a score ranging from 0 to 100. Higher scores indicate better perceived usability, with scores above 68 considered above average and scores above 80 considered excellent^73^. The UEQ (User Experience Questionnaire)^46^ is a standardized questionnaire used to assess the user experience of products, systems, or services. A total of 26 items measure six key dimensions: Attractiveness, Perspicuity (Clarity), Efficiency, Dependability, Stimulation, and Novelty. Participants rate each item on a seven-point scale, ranging from -3 (extremely bad) to + 3 (extremely good). Mean values for each domain are analyzed more frequently than generating an overall score. Values > 0.8 are considered a positive evaluation^74^. In addition, we used a customized version of the UEQ for each test to ensure more precise feedback concerning the difficulty and speed level of each test as well as the perceived informative value of the test.

The testing process followed an iterative design, allowing for step-wise refinement based on feedback from each testing iteration ensuring that it met the specific needs and usability requirements of PD PwPD and enhanced its overall effectiveness in cognitive assessment.

### Analysis and feature extraction

Data analysis was conducted using Python V.3.10 and R V4.2.3. The Jupyter notebook is available on Github [https://github.com/tfeige91/DiCo-iOS].

All analyses were performed using complete cases for the respective analysis. DiCo-based structural analyses were restricted to participants who completed the digital assessment. Correlations with neuropsychological tests and questionnaires were calculated only for participants with available data in the respective modality. Longitudinal analyses were restricted to participants with available baseline and follow-up assessments. Missing data across DiCo, neuropsychological, questionnaire, and follow-up modalities were not imputed.

54 features were extracted from the 13 tests, including mean and standard deviation of the reaction time, percentage of correct answers and others. A full list of features can be found in the supplementary text. To mitigate the risk of multicollinearity, we identified pairs of correlations with a threshold of 0.85 and randomly excluded one variable. A total of four variables from four different tests were thereby excluded from the analysis: flanker_rt_mean_congruent, bart_correctedTotal, kirby_expValue, and StopSignal_correct_go.

Furthermore, we clipped features at +/− 3 * interquartile range to minimize the influence of extreme outliers.

Next, we calculated Variance Inflation Factors (VIFs) separately for each test and excluded variables with a VIF > 10. This test-wise approach ensured that at least one representative variable from each task remained for subsequent structural data analyses, including cluster mapping, network analysis, and exploratory factor analysis.

### Correlations and networks

To detect groups of variables that cohere based on shared variance, we performed a hierarchical agglomerative cluster analysis on the pairwise Pearson correlation matrix of all DiCo-derived variables using average linkage as the linkage criterion and 1 – |r| as the distance metric. The resulting dendrogram and clustered heatmap were generated using the clustermap function from the Seaborn package (version 0.13.2) in Python. This approach groups variables with similar correlation profiles, allowing an intuitive identification of variable clusters that reflect shared cognitive domains or common measurement characteristics. To identify robust relationships between the test variables, we calculated the sparse inverse covariance matrix (precision matrix) using the GraphicalLassoCV function from scikit-learn in Python using default parameters. The sparse inverse covariance matrix reveals information about the conditional dependency of two variables controlling for the effects of all other variables in the dataset. We used the non-diagonal values of the precision matrix to draw a network graph in Gephi, utilizing the force-directed layout algorithm ForceAtlas2^75^. The following tuning parameters were used in the depiction of the final graph: Scaling = 8.0, Gravity = 0.015, Stronger gravity = active.

### Factor analysis

To identify the underlying latent dimensions underlying the observed covariance structure of DiCo features, we performed Exploratory Factor Analysis (EFA). Calculating the Kaiser–Meyer–Olkin (KMO) measure of sampling adequacy (MSA) yielded an unsatisfactory value of < 0.60. We therefore followed the method proposed by Kaiser and Rice^76^ and calculated the MSA for each variable separately, excluding those having an MSA < 0.50. This procedure led to the exclusion of 24 features, leaving 30 features. No variable from Simple Reaction, *Reversal Learning*, *Kirby* and *BART* remained in the EFA dataset. We then performed EFA with a maximum likelihood estimation and oblique (Promax) rotation. The decision on *f*, the number of factors to estimate, was based on the factors’ eigenvalues, keeping those with an eigenvalue > 1.0, which is the commonly used Kaiser’s criterion^47^ in factor analysis. We performed EFA and calculated factor scores using the open-source Python module factor_analyzer^77^ version 0.5.0.

Following common best practices^78^ we then excluded variables with communalities < 0.40 and repeated the EFA using the same extraction procedure described above.

### Clustering

We performed latent profile analysis (LPA) on the factor scores to identify participant-level cognitive performance profiles. LPA was chosen because it is a person-centered, model-based approach that classifies individuals into profiles based on their multivariate pattern of factor scores. Compared with traditional non-latent clustering methods, such as k-means or hierarchical clustering, LPA treats profile membership as an unobserved categorical variable and assigns individuals to profiles based on posterior probabilities estimated directly from the model.^79^ We estimated solutions with different numbers of profiles and selected the final solution based on a combination of Akaike Information Criterion (AIC) or Bayesian Information Criterion (BIC) as well as other measures like entropy, i.e. the goodness of cluster separation (1.0 being the best) and Bootstrap Likelihood Ratio Test (BLRT) that—if significant—suggests the superiority of the k-cluster solution over the k-1-cluster solution. We performed LPA with the R-package tidyLPA^80^. To determine if there are differences between the clusters, we performed a Kruskal–Wallis test followed by Dunn’s test.

### Predicting screening assessments and feature importance

We trained a Random Forest regressor using scikit-learn to predict both MoCA and FAB scores using the factor scores derived from the EFA (as described above), as well as age, age at initial diagnosis, disease duration and LEDD. RF is a robust method known for its efficacy in handling complex, non-linear relationships among variables.^81^ Hyperparameters were optimized via grid search with fivefold cross-validation, minimizing the mean absolute error (MAE). Model performance was then evaluated using repeated 5 × twofold cross-validation, with three different random seeds per split to account for variance introduced by both data partitioning and the stochastic nature of the algorithm. For each model, the MAE and root mean squared error (RMSE) were recorded on held-out folds. Feature importances were averaged across all runs, and we additionally quantified their standard deviation due to random forest construction and cross-validation splits. Because hyperparameter tuning was not embedded in a fully nested cross-validation framework, performance estimates may be slightly optimistic and were interpreted descriptively.

### Longitudinal analysis

A total of 28 PwPD completed follow-up assessments using a subset of 6 DiCo tests: *Simple Reaction Time Test, Stroop Test, N-Back, Tower of London, Flanker, and the Judgement of Line Orientation Task*. The average interval between baseline and follow-up was 770 days (SD = 236). To evaluate cognitive change over time, we calculated follow-up factor scores for *Working Memory*, *Interference Control,* and *Problem-Solving*. These were derived using the factor loadings established from the baseline sample and restricted to features from the subset of tasks available at both timepoints. Correlations between these follow-up scores and the full-model factor scores (using the complete feature set) exceeded r = 0.95, confirming the robustness of the reduced-score estimation. We calculated Pearson correlation coefficients to assess the relationship between the change in the MoCA score and the time between baseline and follow-up.

### Features responsive to longitudinal change

To quantify the magnitude of longitudinal change, we calculated the Standardized Response Mean (SRM) for each cognitive domain. The SRM is defined as the mean change score between baseline and follow-up divided by the standard deviation of the change scores. It represents an effect size metric commonly used to evaluate sensitivity to change, with values around 0.2, 0.5, and 0.8 typically interpreted as small, moderate, and large effects, respectively.

### Calculation of a continuous motor score

To derive an exploratory continuous motor score that integrates multiple medication-related and clinical variables, we applied Uniform Manifold Approximation and Projection (UMAP)^82,83^, a non-linear dimensionality reduction technique that preserves the local and global structure of high-dimensional data in a low-dimensional embedding. This exploratory composite summarized motor symptom severity and dopaminergic treatment burden in a single variable, acknowledging that UPDRS III scores may be influenced by medication state and treatment adjustments. We selected four variables reflecting motor severity and medication status: MDS-UPDRS III motor score, levodopa equivalent daily dose from dopamine agonists (LEDD_DA), levodopa equivalent daily dose from levodopa (LEDD_L), and total LEDD. Baseline and follow-up observations were concatenated to embed both timepoints in the same low-dimensional space. The UMAP algorithm was applied to the z-standardized variables with the following parameters: n_components = 1, n_neighbors = 30, and random_state = 24. The resulting one-dimensional embedding was then min–max scaled to a range between 0 and 1, with higher values indicating more severe motor impairment. Since UMAPs do not yield explicit feature weights, we calculated pairwise Pearson correlation coefficients between the input features and the UMAP-derived motor score to assess their individual contributions (Supplementary Fig. 5).

We conducted paired t-tests on both the UPDRS III and the UMAP-derived Motor Score to assess whether significant changes occurred between baseline and follow-up assessments.

### Identifying baseline features to predict cognitive and motor progression

To exploratorily identify relevant features for predicting the change in both MoCA and the motor score we calculated feature importances using the same Random Forest Regressor algorithm described above.

### Relation of cognitive and motor changes

To examine the relationship between cognitive and motor changes over time, we calculated the change scores (Δ) for the MoCA and the UMAP-derived motor score by subtracting baseline values from follow-up values. To control for baseline effects, we performed linear regression analyses by regressing follow-up scores on baseline scores for each measure. The residuals from these regressions reflect change independent of baseline performance. We then computed the Pearson correlation between these residuals to assess the association between motor and cognitive change.

To further investigate the role of reflection impulsivity and medication in motor progression, we conducted two linear regression analyses. First, we tested whether baseline Decision-Making performance predicted changes in motor functioning (Δ Motor Score). Second, we examined the association between the proportion of dopamine agonists in the total levodopa equivalent dose at baseline (DA/LEDD Total) and motor progression. In both models, we estimated a linear regression line and computed Pearson correlation coefficients to quantify the strength of the relationship. Results were visualized using regression plots with 95% confidence intervals.

## Supplementary Information

Supplementary Information.

## Data Availability

Data is provided within the manuscript or supplementary information files.
