# Supplementary material for: Cognitive structure and progression in Parkinson’s disease: insights from a tablet-based assessment
Source: Sci Rep. 2026 Jul 15;16:22255. doi: 10.1038/s41598-026-62324-6 (PMC13373175; doi:10.1038/s41598-026-62324-6)
Supplement: Supplementary file 1 — Supplementary Information. [file 41598_2026_62324_MOESM1_ESM.pdf]

Cognitive structure and progression in Parkinson's Disease: Insights from a tablet-based assessment

- Supplementary Material -

Tim Feige[1,2,*], Anika Frank[1,2,*], Jonas Bendig[1], Andrea Epler[1], Charlotte Harbarth[1], Julia Kunze[1], Johanna Jungk[1], Nils Schnalke[1], Heinz Reichmann[1], Björn H. Falkenburger[1,2]

1) Department of Neurology, Faculty of Medicine and University Hospital Carl Gustav Carus, Technische Universität Dresden, Dresden, Germany
2) German Center for Neurodegenerative Diseases (DZNE), Dresden, Germany
*) Tim Feige and Anika Frank contributed equally to this work

Corresponding author:
Prof. Björn H. Falkenburger
Department of Neurology, University Hospital Carl Gustav Carus
Technische Universität Dresden
Fetscherstrasse 74, 01307 Dresden, Germany

Contents:

Supplementary Table 1: Measures of central tendencies and split-half reliability
Supplementary Table 2: Latent Profile analysis to determine number of clusters

Supplementary Fig. 1 Inverting time-based Variables in C1 leads to two clusters
Supplementary Fig. 2 Correlations between features and UMAP-derived motor score
Supplementary Fig. 3 Correlation between motor function and reaction time
Supplementary Fig. 4 Individual correlations between EFA factors and NPT results

Overview DiCo Tests

Supplementary Table 1 Measures of central tendencies and split-half reliability of all DiCo derived features

Feature	Mean (SD)	Median	Range	Split-Half-Reliability
**DiCo**				
**Simple Reaction**[17]				
Mean reaction time	469.21 (127.65)	428.94	[282.24–1010.51]	.98 [.97-.99]
Reaction time sd	105.28 (72.35)	79.98	[24.84–394.91]	.82 [.75-.86]
Reaction time trend	-0.94 (2.05)	-0,81	[-6.11–6.79]	-
**Stroop**[17]				
N correct in congruent trials	0.91 (0.2)	1	[0.0–1.0]	.96 [.94-.97]
N correct in incongruent trials	0.71 (0.35)	0.88	[0.0–1.0]	.97 [.95-.98]
Reaction time congruent trials	775.82 (164.7)	805.62	[114.21–1087.79]	.89 [.84-.93]
Reaction time incongruent trials	738.03 (249.82)	809.29	[-1.0–1127.62]	.91 [.88-.94]
Reaction time sd congruent trials	226.74 (133.65)	186.76	[71.16–627.19]	.82 [.73-.89]
Reaction time sd incongruent trials	330.71 (174.51)	314.39	[0.0–670.27]	.88 [.82-.92]
**Go-Nogo**[17]				
N correct in go trials	0.94 (0.09)	0.98	[0.44–1.0]	.96 [.93-.97]
N correct in no-go trials	0.94 (0.07)	0.97	[0.63–1.0]	.73 [.61-.82]
Reaction time go trials	465.5 (61.35)	461.14	[334.24–646.97]	.96 [.94-.97]
Reaction time sd go trials	81.97 (31.57)	75.04	[48.7–244.23]	.96 [.94-.97]

Feature	Mean (SD)	Median	Range	Split-Half-Reliability
**Baloon Analogue Risk Task**[58]				
Pumps	0.54 (0.22)	0.51	[0.15–1.12]	.82 [.74-.87]
Change after burst occured	-2.53 (3.1)	-2.5	[-11.67–7.12]	-.04 [-.81-.51]
% Bursts	0.18 (0.08)	0.17	[0.0–0.37]	.38 [.22-.53]
Total Points	8.44 (3.09)	8.10	[2.35–17.53]	.33 [-.02-.58]
**NBack**[17]				
N correct n=1 trials	37.33 (12.77)	43	[1.0–50.0]	.88 [.82-.92]
N correct n=2 trials	24.77 (11.28)	27	[1.0–45.0]	.95 [.93-.96]
Reaction time n=1 trials	796.61 (159.89)	796.38	[370.88–1248.26]	.97 [.95-.98]
Reaction time n=2 trials	698.05 (272.69)	710.38	[21.2–1286.84]	.94 [.91-.96]
Reaction time sd n=1 trials	371.27 (203.07)	305.81	[100.26–929.44]	.88 [.84-.91]
Reaction time sd n=2 trials	460.96 (190.18)	473.99	[121.64–864.65]	.92 [.90-.94]
**Tower of London**[17]				
Stimulus Duration (time needed)	5993.9 (2519.54)	5337.33	[2671.92–14968.83]	.83 [.77-.87]
% correctly solved problems	0.53 (0.27)	0.58	[0.0–1.0]	.79 [.72-.84]
**Kirby**[17]				
Exponential discount rate value	0.05 (0.06)	0.02	[0.0–0.14]	.72 [.64-.80]
Hyperbolic discount rate value	0.09 (0.1)	0.03	[0.0–0.25]	.74 [.67-.81]

Feature	Mean (SD)	Median	Range	Split-Half-Reliability
**Flanker**[17]				
% correct in congruent trials	0.98 (0.06)	1	[0.6–1.0]	.74 [.50-.89]
% correct in incongruent trials	0.97 (0.06)	1	[0.73–1.0]	.60 [.31-.77]
Reaction time congruent trials	873.88 (299.3)	815	[499.44–2441.0]	.93 [.90-.96]
Reaction time incongruent trials	959.32 (278.04)	897	[563.29–1858.29]	.90 [.85-.94]
Reaction time sd congruent trials	224.96 (192.22)	157.74	[46.02–907.01]	.78 [.69-.85]
Reaction time sd incongruent trials	247.51 (218.82)	153.36	[37.5–1079.73]	.80 [.73-.86]
**Stop Signal**[17]				
N correct in all trials	0.84 (0.07)	0.86	[0.51–0.94]	.75 [.67-.81]
N correct in go trials	0.94 (0.08)	0.97	[0.44–1.0]	.90 [.86-.93]
N correct in stop trials	0.59 (0.12)	0.6	[0.13–0.8]	.44 [.29-.59]
Max delay value in high condition	491.24 (142.16)	500	[250.0–850.0]	.99 [.98-.99]
Max delay value in low condition	410.82 (100.06)	400	[250.0–650.0]	.99 [.99-.99]
Reaction time in go trials	673.96 (128.57)	677.59	[306.33–965.4]	.96 [.94-.97]
Reaction time sd in go trials	198.09 (86.09)	177.5	[75.32–587.12]	.89 [.85-.92]
**Reversal Learning**[59]				
N correct in all trials				
Change after probabilistic feedback				

Feature	Mean (SD)	Median	Range	Split-Half-Reliability
N probabilistic errors				
N reversal errors				
N total trials				
Ratio changes/PWF feedback				
**Judgement of Line Orientation**[60]				
Reaction time	9788.93 (4412.66)	8327.79	[3707.0–33820.17]	.93 [.90-.96]
% correct	0.69 (0.21)	0.73	[0.0–1.0]	.87 [.82-.90]
Mean Distance to correct answer	0.38 (0.31)	0.3	[0.0–2.0]	.74 [.63-.83]
**Information Sampling**[17]				
Reaction time decreased win	1423.51 (704.83)	1284.59	[357.54–3795.32]	.78 [.66-.85]
Reaction time fixed win	1378.33 (826.05)	1130.2	[352.69–5153.43]	.88 [.78-.94]
N clicks decreased win	8.63 (4.51)	8	[0.89–21.67]	.93 [.90-.95]
N clicks fixed win	11.88 (6.47)	45668	[0.2–25.0]	.97 [.95-.98]

Supplementary Table 2 Associations between DiCo factor scores and neuropsychological test performance

Feature	Visual Attention				Interference Control				Problem-Solving			
	r	CI	p	n	r	CI	p	n	r	CI	p	n
CERAD ANIM (r)	.33	[.06, .56]	.02	50	.44	[.18, .64]	<.01	50	.48	[.23, .67]	<.01	50
CERAD TMT-B (r)	-.34	[-.57, -.07]	.02	49	-.64	[-.78, -.44]	<.01	49	-.52	[-.70, -.28]	<.01	49
BTA Total (r)	.31	[.03, .54]	.03	50	.50	[.26, .68]	<.01	50	.35	[.08, .58]	.01	50
BTA NUM (r)	.26	[-.02, .50]	.07	50	.41	[.14, .61]	<.01	50	.40	[.13, .61]	<.01	50
WIE (r)	.36	[.09, .58]	.01	50	.41	[.15, .62]	<.01	50	.32	[.05, .55]	.02	50
CERAD FIGSAV (r)	.24	[-.04, .49]	.09	50	.41	[.14, .62]	<.01	50	.25	[-.03, .50]	.08	50
WMSR DS-B (r)	.23	[-.05, .48]	.11	50	.28	[.00, .52]	.05	50	.25	[-.03, .50]	.08	50
CERAD FIGR (r)	.22	[-.06, .47]	.13	50	.43	[.18, .63]	<.01	50	.27	[-.01, .51]	.06	50
CERAD WL3 (r)	.19	[-.09, .45]	.18	50	.36	[.10, .58]	<.01	50	.28	[.00, .52]	.05	50
CERAD WLREC (r)	.18	[-.11, .43]	.22	50	.33	[.06, .56]	.02	50	.28	[.00, .51]	.05	50
BTA LET (r)	.21	[-.07, .46]	.14	50	.40	[.14, .61]	<.01	50	.21	[-.07, .46]	.14	50
CERAD MMSE (r)	.20	[-.09, .45]	.17	50	.33	[.06, .56]	.02	50	.36	[.10, .58]	<.01	50
CERAD TMT-A (r)	-.08	[-.36, .20]	.57	49	-.41	[-.62, -.14]	<.01	49	-.32	[-.55, -.04]	.02	49
CERAD WLTOT (r)	.16	[-.12, .42]	.26	50	.37	[.10, .59]	<.01	50	.24	[-.04, .49]	.09	50
CERAD WL2 (r)	.16	[-.13, .42]	.28	50	.35	[.08, .57]	.01	50	.30	[.02, .53]	.03	50
/MSR DS-Total (r)	.19	[-.09, .45]	.18	50	.21	[-.07, .46]	.15	50	.14	[-.14, .40]	.33	50
MWT-B (r)	.13	[-.15, .39]	.37	50	-.06	[-.33, .22]	.67	50	-.20	[-.45, .09]	.17	50
VOSP (r)	.09	[-.19, .36]	.53	50	.23	[-.05, .48]	.10	50	.19	[-.09, .45]	.19	50

CERAD INTRU (r)	-.22	[-.47, .06]	.12	50	-.29	[-.52, -.01]	.04	50	.01	[-.26, .29]	.92	50
CERAD WLRECog (r)	.01	[-.27, .29]	.95	50	.23	[-.05, .48]	.11	50	.08	[-.20, .35]	.57	50
CERAD BNT (r)	.14	[-.14, .41]	.32	50	.06	[-.22, .34]	.66	50	.00	[-.28, .27]	.97	50
CERAD WL1 (r)	.10	[-.18, .37]	.48	50	.32	[.04, .55]	.03	50	.09	[-.19, .36]	.54	50
CERAD TMT-Ratio (r)	-.20	[-.46, .08]	.17	49	-.05	[-.33, .23]	.72	49	-.11	[-.38, .18]	.46	49
CERAD SW (r)	.02	[-.26, .30]	.88	50	.17	[-.11, .43]	.23	50	.07	[-.21, .35]	.61	50
CERAD FIGC (r)	.01	[-.27, .29]	.94	50	.22	[-.07, .47]	.13	50	.16	[-.13, .42]	.27	50
WMSR DS-F (r)	.05	[-.23, .32]	.74	50	.05	[-.23, .32]	.74	50	-.05	[-.33, .23]	.72	50
CERAD WLSAV (r)	-.04	[-.31, .25]	.80	49	.01	[-.27, .29]	.94	49	.03	[-.26, .31]	.86	49

Supplementary Table 2 continued

Feature	Decision-Making				Working Memory			
	r	CI	p	n	r	CI	p	n
CERAD ANIM (r)	.17	[-.11, .43]	.24	50	.43	[.17, .63]	<.01	50
CERAD TMT-B (r)	.02	[-.26, .30]	.90	49	-.21	[-.47, .07]	.14	49
BTA Total (r)	.19	[-.09, .44]	.19	50	.29	[.01, .52]	.04	50
BTA NUM (r)	.18	[-.10, .44]	.20	50	.32	[.05, .55]	.02	50
WIE (r)	.19	[-.09, .44]	.19	50	.20	[-.08, .45]	.16	50
CERAD FIGSAV (r)	-.09	[-.36, .20]	.56	50	.34	[.07, .56]	.02	50
WMSR DS-B (r)	.33	[.05, .56]	.02	50	.20	[-.08, .46]	.16	50
CERAD FIGR (r)	-.06	[-.33, .22]	.69	50	.28	[.00, .51]	.05	50

CERAD WL3 (r)	.13	[-.15, .39]	.37	50	.25	[-.03, .50]	.08	50
CERAD WLREC (r)	.09	[-.19, .36]	.52	50	.29	[.01, .52]	.04	50
BTA LET (r)	.17	[-.11, .43]	.24	50	.14	[-.14, .40]	.33	50
CERAD MMSE (r)	.00	[-.28, .28]	1.00	50	.14	[-.15, .40]	.34	50
CERAD TMT-A (r)	-.12	[-.39, .17]	.42	49	-.06	[-.34, .23]	.68	49
CERAD WLTOT (r)	.06	[-.23, .33]	.70	50	.15	[-.13, .41]	.29	50
CERAD WL2 (r)	.04	[-.24, .32]	.78	50	.11	[-.17, .38]	.45	50
/MSR DS-Total (r)	.27	[-.01, .51]	.06	50	.14	[-.14, .40]	.33	50
MWT-B (r)	.37	[.11, .59]	<.01	50	.16	[-.12, .42]	.25	50
VOSP (r)	.25	[-.04, .49]	.09	50	-.07	[-.35, .21]	.61	50
CERAD INTRU (r)	-.10	[-.37, .18]	.50	50	-.11	[-.37, .18]	.46	50
CERAD WLRECog (r)	.28	[.01, .52]	.05	50	.11	[-.18, .38]	.45	50
CERAD BNT (r)	.32	[.05, .55]	.02	50	.10	[-.19, .37]	.50	50
CERAD WL1 (r)	-.02	[-.30, .26]	.88	50	.06	[-.23, .33]	.70	50
CERAD TMT-Ratio (r)	.15	[-.13, .42]	.29	49	-.05	[-.33, .23]	.73	49
CERAD SW (r)	.02	[-.26, .30]	.88	50	.25	[-.03, .50]	.08	50
CERAD FIGC (r)	.02	[-.26, .30]	.87	50	-.02	[-.30, .26]	.87	50
WMSR DS-F (r)	.16	[-.12, .42]	.26	50	-.03	[-.30, .25]	.84	50
CERAD WLSAV (r)	-.04	[-.31, .25]	.80	49	.11	[-.17, .38]	.44	49

Supplementary Table 3 Associations between DiCo factor scores and questionnaire data

Feature	Visual Attention				Interference Control				Problem-Solving			
	r	CI	p	n	r	CI	p	n	r	CI	p	n
PGI Severity	-.35	[-.53, -.14]	<.01	77	-.36	[-.54, -.15]	<.01	77	-.46	[-.62, -.26]	<.01	77
UPPS Lack Persev.	-.44	[-.60, -.24]	<.01	79	-.28	[-.47, -.07]	.01	79	-.38	[-.55, -.17]	<.01	79
UPPS Urgency	-.27	[-.46, -.05]	.02	79	-.26	[-.45, -.04]	.02	79	-.25	[-.45, -.03]	.03	79
BFI Conscientiousness	-.47	[-.63, -.28]	<.01	80	-.34	[-.52, -.13]	<.01	80	-.23	[-.43, -.01]	.04	80
FAQ Total	-.45	[-.61, -.26]	<.01	80	-.25	[-.45, -.03]	.02	80	-.25	[-.45, -.04]	.02	80
PDQ-8 Total	-.31	[-.50, -.10]	<.01	80	-.33	[-.51, -.12]	<.01	80	-.32	[-.50, -.11]	<.01	80
OCI-R Total	-.33	[-.52, -.12]	<.01	77	-.26	[-.46, -.04]	.02	77	-.24	[-.44, -.02]	.04	77
Apathy	-.31	[-.49, -.09]	<.01	80	-.27	[-.47, -.06]	.01	80	-.31	[-.50, -.10]	<.01	80
ICDRC Gambling	-.30	[-.50, -.09]	<.01	76	-.33	[-.52, -.12]	<.01	76	-.12	[-.34, .11]	.30	76
Fatigue (FSS)	-.33	[-.55, -.07]	.01	53	-.34	[-.56, -.08]	.01	53	-.28	[-.51, -.01]	.04	53
ICDRC Shopping	-.35	[-.53, -.13]	<.01	76	-.26	[-.46, -.04]	.02	76	-.23	[-.43, .00]	.05	76
EQ-5D Index	.25	[.03, .44]	.03	80	.30	[.09, .49]	<.01	80	.26	[.05, .46]	.02	80
BIS-11 Attention	-.15	[-.36, .08]	.20	77	-.25	[-.45, -.03]	.03	77	-.34	[-.53, -.13]	<.01	77
BFI Openness	-.25	[-.44, -.03]	.03	80	-.21	[-.41, .01]	.06	80	-.22	[-.42, .00]	.05	80
PDSS Total	-.20	[-.40, .02]	.07	80	-.16	[-.37, .06]	.15	80	-.30	[-.49, -.09]	<.01	80

MS-Quest	-.24	[-.44, -.03]	.03	81	-.16	[-.36, .07]	.17	81	-.34	[-.52, -.13]	<.01	81
ESS Total	-.16	[-.36, .06]	.15	82	-.20	[-.40, .02]	.07	82	-.29	[-.47, -.07]	<.01	82
BIS-11 Total	-.27	[-.47, -.05]	.02	77	-.13	[-.35, .10]	.25	77	-.22	[-.42, .00]	.05	77
ICDRC Repetitive	-.22	[-.42, .01]	.06	76	-.09	[-.31, .13]	.42	76	-.19	[-.40, .04]	.11	76
BIS-11 Nonplanning	-.33	[-.52, -.12]	<.01	77	-.08	[-.30, .15]	.49	77	-.21	[-.42, .01]	.07	77
Depression (BDI)	-.17	[-.37, .05]	.14	80	-.19	[-.39, .04]	.10	80	-.19	[-.39, .03]	.09	80
QUIP-RS Total	-.12	[-.34, .10]	.29	77	-.10	[-.31, .13]	.41	77	-.16	[-.37, .07]	.17	77
ICDRC DDS	-.14	[-.36, .08]	.21	76	-.28	[-.48, -.06]	.01	76	.01	[-.22, .23]	.96	76
BFI Extraversion	-.15	[-.36, .07]	.17	80	-.15	[-.36, .07]	.19	80	-.17	[-.38, .05]	.13	80
UPPS Sensat. Seek.	-.04	[-.26, .18]	.72	79	.14	[-.08, .35]	.20	79	.19	[-.04, .39]	.10	79
BFI Agreeableness	.00	[-.22, .22]	.98	80	-.06	[-.27, .17]	.62	80	.14	[-.08, .35]	.21	80
ICDRC Sexuality	.05	[-.17, .27]	.65	76	.12	[-.11, .34]	.29	76	.12	[-.11, .34]	.29	76
UPPS Lack Premed.	-.05	[-.26, .18]	.69	79	.17	[-.05, .38]	.13	79	.00	[-.22, .22]	1.00	79
EQ-5D VAS	-.03	[-.25, .19]	.80	80	-.08	[-.30, .14]	.46	80	-.03	[-.24, .20]	.82	80
BFI Emotional Stability	.06	[-.17, .27]	.62	80	.06	[-.17, .27]	.62	80	-.06	[-.27, .16]	.61	80
ICDRC Eating	-.04	[-.26, .19]	.75	76	-.02	[-.24, .21]	.88	76	-.05	[-.27, .18]	.65	76
BIS-11 Motor	-.12	[-.34, .10]	.28	77	-.02	[-.25, .20]	.84	77	.00	[-.23, .22]	.98	77
PGI Change	.08	[-.14, .30]	.47	76	.04	[-.19, .26]	.74	76	-.02	[-.24, .21]	.87	76

Supplementary Table 3 continued

Feature	Decision-Making				Working Memory			
	r	CI	p	n	r	CI	p	n
PGI Severity	-.17	[-.38, .06]	.15	77	-.14	[-.35, .09]	.22	77
UPPS Lack Persev.	.01	[-.21, .24]	.90	79	-.28	[-.48, -.07]	.01	79
UPPS Urgency	-.28	[-.47, -.07]	.01	79	-.34	[-.52, -.13]	<.01	79
BFI Conscientiousness	-.16	[-.36, .07]	.17	80	-.15	[-.36, .08]	.19	80
FAQ Total	-.16	[-.37, .06]	.16	80	-.22	[-.42, .00]	.05	80
PDQ-8 Total	-.12	[-.33, .11]	.31	80	-.19	[-.39, .03]	.09	80
OCI-R Total	-.23	[-.43, .00]	.05	77	-.15	[-.37, .07]	.18	77
Apathy	-.14	[-.35, .08]	.20	80	-.17	[-.37, .05]	.14	80
ICDRC Gambling	-.20	[-.41, .03]	.09	76	-.19	[-.40, .03]	.09	76
Fatigue (FSS)	-.01	[-.28, .26]	.95	53	-.13	[-.39, .14]	.35	53
ICDRC Shopping	-.08	[-.30, .15]	.51	76	-.14	[-.35, .09]	.23	76
EQ-5D Index	-.06	[-.27, .17]	.62	80	.18	[-.04, .38]	.11	80
BIS-11 Attention	-.06	[-.28, .16]	.59	77	-.24	[-.44, -.01]	.04	77
BFI Openness	-.23	[-.43, -.01]	.04	80	-.13	[-.34, .09]	.24	80
PDSS Total	.21	[-.01, .41]	.06	80	-.15	[-.35, .08]	.20	80
MS-Quest	.15	[-.07, .36]	.18	81	-.05	[-.27, .17]	.63	81
ESS Total	.15	[-.07, .35]	.18	82	-.08	[-.29, .14]	.47	82

BIS-11 Total	-.04	[-.27, .18]	.71	77	-.09	[-.31, .14]	.44	77
ICDRC Repetitive	.17	[-.06, .38]	.14	76	-.03	[-.25, .20]	.82	76
BIS-11 Nonplanning	-.02	[-.24, .21]	.88	77	-.05	[-.27, .18]	.69	77
Depression (BDI)	-.04	[-.26, .18]	.71	80	-.10	[-.31, .13]	.40	80
QUIP-RS Total	-.05	[-.27, .17]	.65	77	-.21	[-.42, .01]	.06	77
ICDRC DDS	-.04	[-.26, .19]	.74	76	-.14	[-.35, .09]	.23	76
BFI Extraversion	.05	[-.17, .27]	.66	80	-.04	[-.26, .18]	.71	80
UPPS Sensat. Seek.	-.01	[-.23, .21]	.95	79	.18	[-.04, .39]	.11	79
BFI Agreeableness	-.23	[-.43, -.01]	.04	80	.03	[-.20, .24]	.83	80
ICDRC Sexuality	.04	[-.19, .26]	.72	76	.11	[-.11, .33]	.32	76
UPPS Lack Premed.	.10	[-.13, .31]	.39	79	.05	[-.17, .27]	.63	79
EQ-5D VAS	-.14	[-.35, .08]	.20	80	-.02	[-.23, .20]	.89	80
BFI Emotional Stability	-.07	[-.29, .15]	.54	80	.03	[-.19, .24]	.82	80
ICDRC Eating	.08	[-.15, .30]	.48	76	.04	[-.18, .27]	.71	76
BIS-11 Motor	-.04	[-.26, .19]	.75	77	.03	[-.20, .25]	.81	77
PGI Change	.06	[-.17, .28]	.63	76	-.01	[-.23, .22]	.96	76

Supplementary Table 4 *Latent Profile analysis to determine number of clusters*

Classes	AIC	BIC	Entropy	prob_min	prob_max	n_min	n_max	BLRT_p
1	1256.6	1382.34	1.00	1.00	1.00	1.00	1.00	0.01
2	1215.79	1256.99	0.96	0.98	0.99	0.18	0.82	0.02
3	1205.84	1262.48	0.91	0.88	0.99	0.13	0.69	0.01
4	1178.98	1251.07	0.88	0.91	0.98	0.10	0.55	0.01
5	1164.31	1251.85	0.86	0.79	0.99	0.10	0.44	0.15

Note. The solution with four clusters was chosen based on BIC, BLRT_p and a reasonably large smallest cluster (n_min).

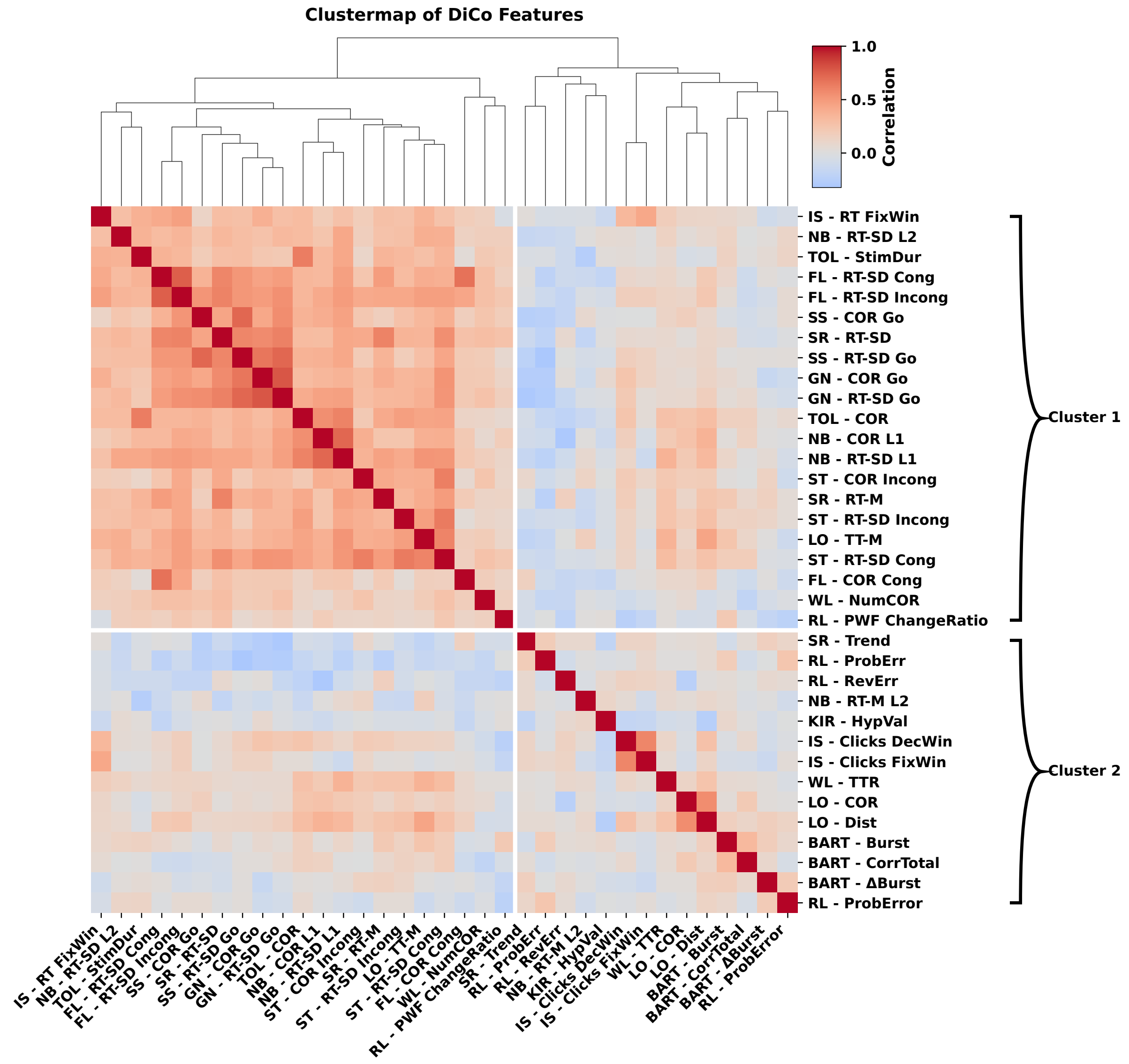

Supplementary Fig. 1 Inverting time-based Variables in C1 leads to two clusters. *C1 includes both reaction time and accuracy-based Variables derived from several tasks. C2 represents a residual group, primarily consisting of features from impulsivity-related tasks.*

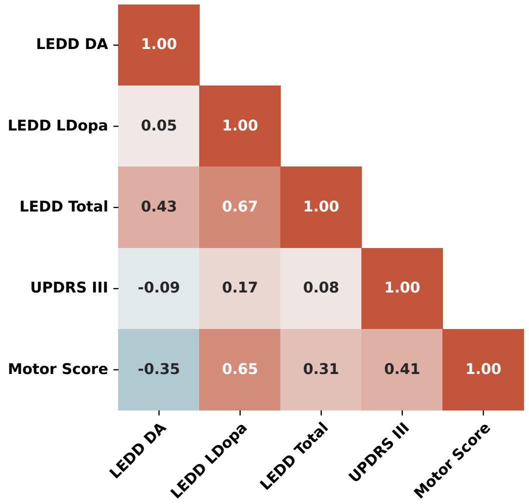

Supplementary Fig. 2 Correlations between features and UMAP-derived motor score. *All features included in the UMAP training except for the dopamine agonist LEDD (LEDD DA) were positively correlated with the UMAP-derived Motor Score (Fig. 9f). The strongest association was observed for the levodopa daily dose (LEDD LDopa) with r = 0.65, followed by UPDRS III (r = 0.41) and total LEDD (r = 0.31). In contrast, dopamine agonist LEDD was negatively associated with the Motor Score (r = −0.35).*

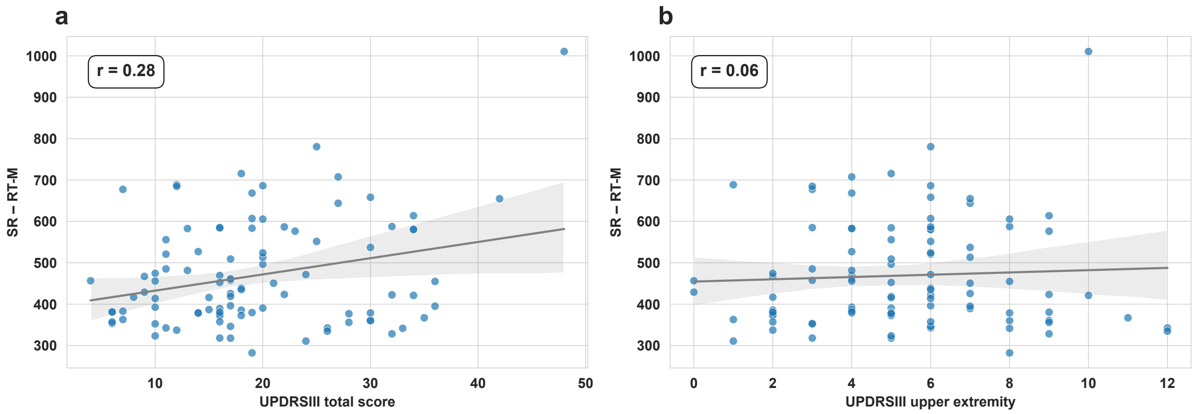

Supplementary Fig. 3 Correlation between motor function and reaction time. *Pearson correlation between UPDRSIII total score (a) and a derived upper extremity motor function score as the sum of UPDRSIII Items 4,5,6 and 16 (b).*

Following pages:

Supplementary Fig. 4 Individual correlations between raw DiCo-derived features and paper pencil neuropsychological tests (a) and questionnaires (b).

Overview DiCo Tests

The DiCo was implemented and conducted in German. German is therefore also the language of the screenshots. Instructions are translated into English in the legend for clarity.

Allgemeine Instruktion

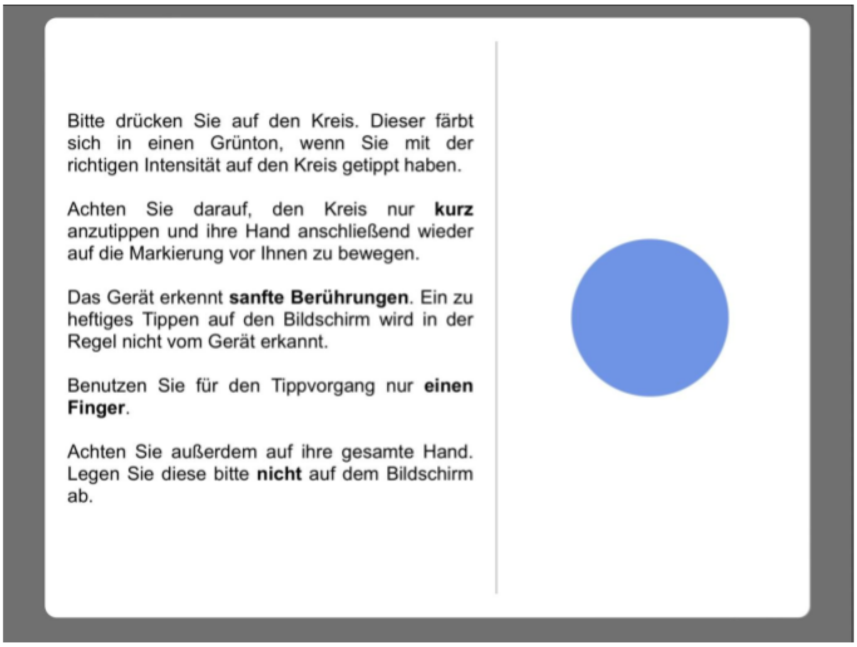

general-instruction

- Übung des Tippverhaltens

Participants see a blue circle. They are instructed to tap on it with one finger. If the tap is successful, the circle turns green; if the patient performs a drag movement, the circle turns red. After five successful taps, the exercise is completed.

Word List

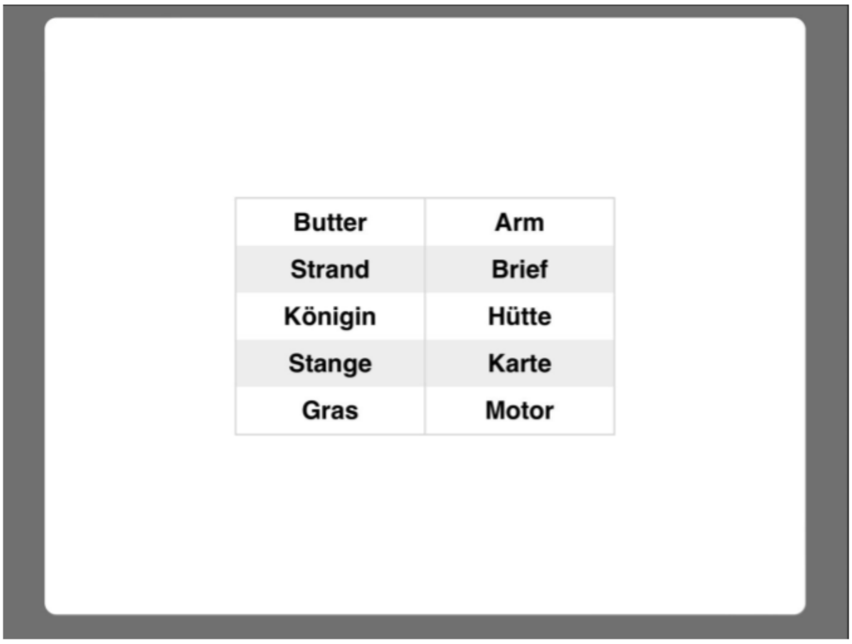

wordlist-one

verbal memory

- Memorizing 10 words, recall at the end of the test battery

Participants are shown ten nouns: "butter", "arm", "beach", "letter", "queen", "hut", "pole", "map", "grass", "motor" in a table (2 columns, 5 rows) for 60 seconds.
At the end of the cognitive battery, the participants are shown an empty table. The participants are asked to type into a cell in the table and enter a word that they have memorized.
The number of memorized words is evaluated. Upper and lower case letters and umlauts are not taken into account.

Simple Reaction Task

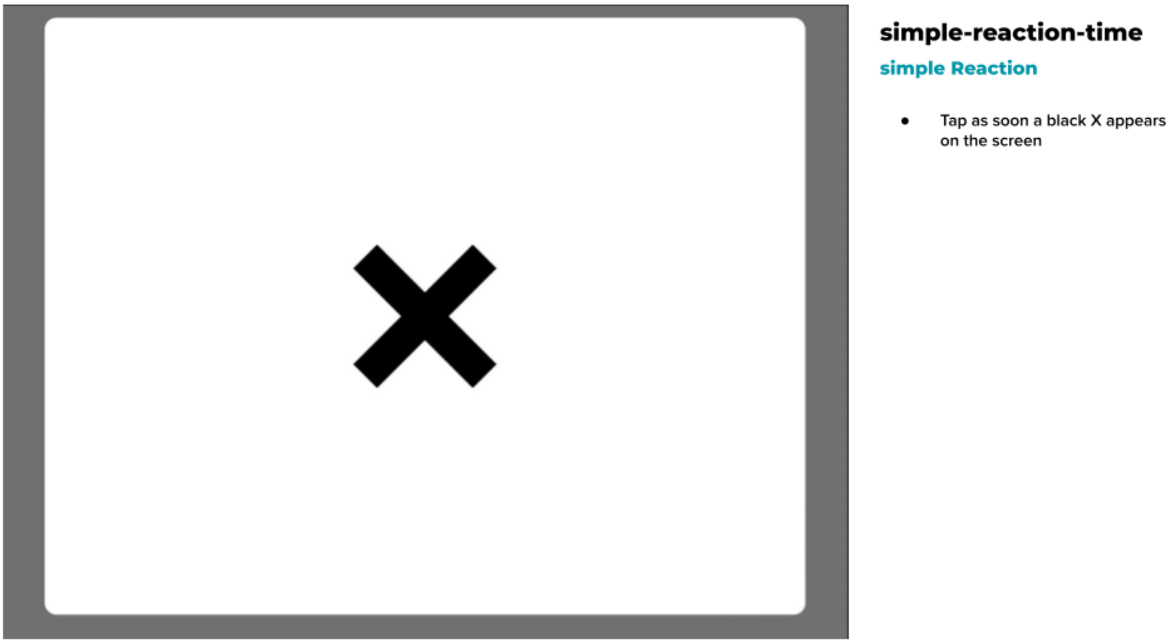

Participants are instructed to tap the screen as soon as they see a black cross. The intervals between individual trials randomly vary between 1200 ms and 2400 ms. After a practice round of five trials, the test round follows with 51 trials

Extracted Features

Variable Name	Description
SimpleReaction_rt_mean	Average reaction time of the participant across all trials.
SimpleReaction_rt_std	Standard deviation of the reaction time across all trials.

Stroop

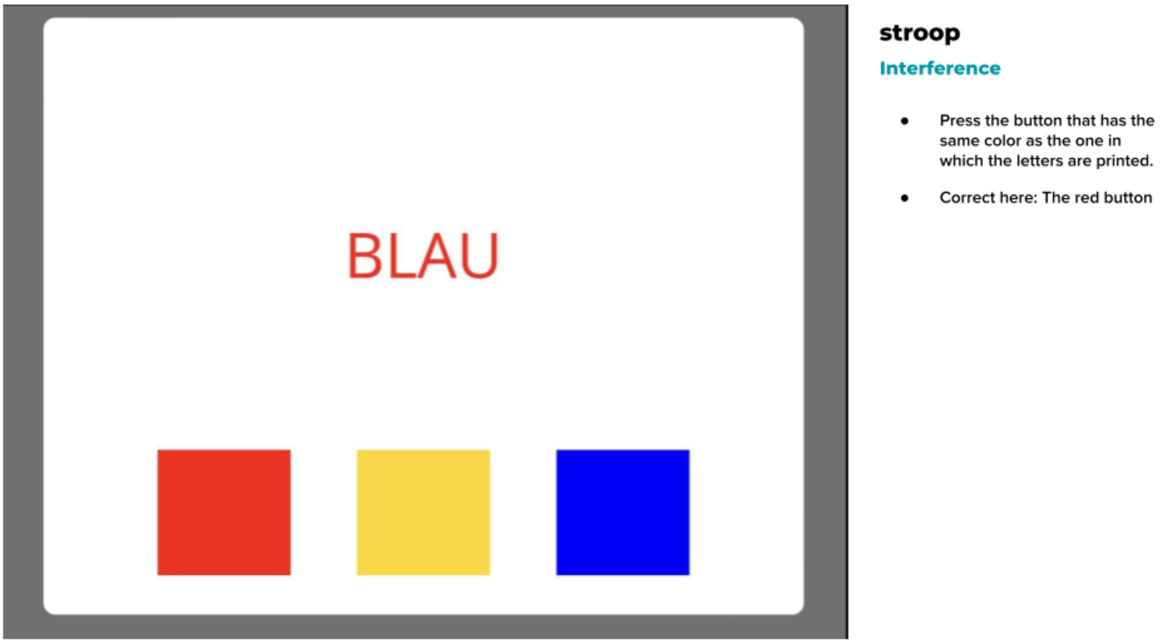

Participants see a displayed color word: “BLUE”, “YELLOW”, “RED”, which is shown either in blue, red, or yellow color. At the bottom, three square buttons in red, yellow, and blue are displayed. Participants are instructed to tap the button that matches the color in which the word is printed after a color word appears. A condition is called “congruent” if the color word matches the color it is printed in. If not, the condition is termed “incongruent”. Initially, there are twelve practice trials: twice each congruent condition and each incongruent condition once. During the practice phase, participants receive feedback on whether the pressed button was correct. After the practice, 48 test trials follow: 4 times each of the six incongruent and 8 times each congruent pairing. There is a 1000 ms interval between each trial.

Extracted Features

Variable Name	Description
Stroop_correct_sum_congruent	Total number of correct responses in congruent conditions.
Stroop_correct_sum_incongruent	Total number of correct responses in incongruent conditions.
Stroop_rt_mean_congruent	Average reaction time for congruent trials.
Stroop_rt_mean_incongruent	Average reaction time for incongruent trials.
Stroop_rt_std_congruent	Standard deviation of reaction time in congruent trials.
Stroop_rt_std_incongruent	Standard deviation of reaction time in incongruent trials.

Go-Nogo

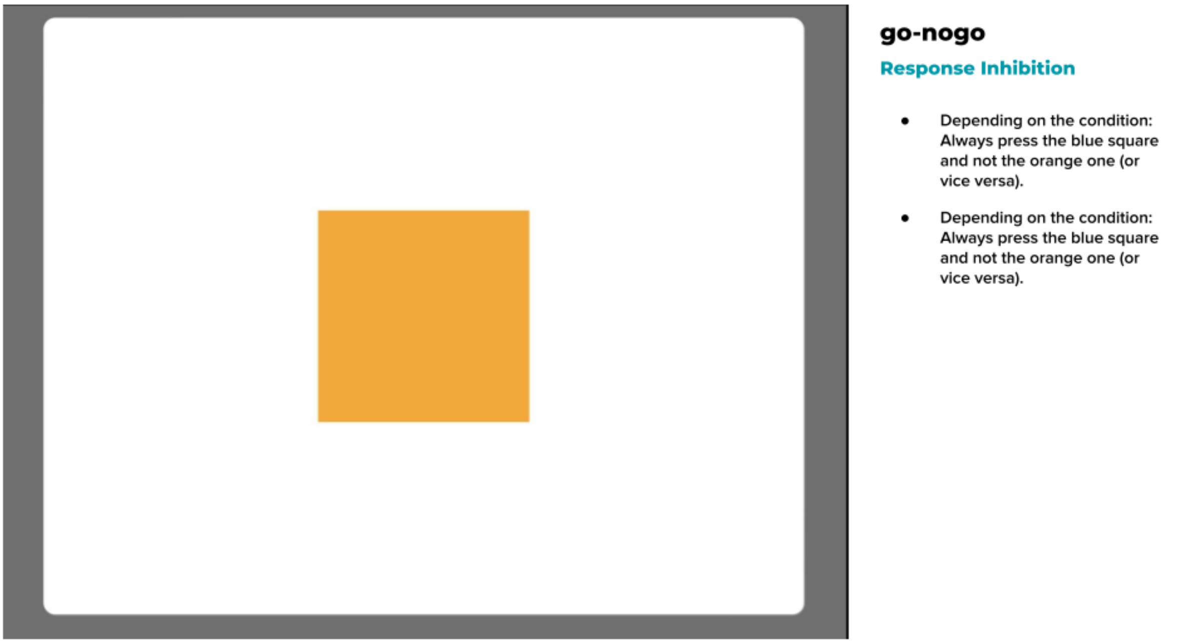

Participants see either a blue or an orange square. They are instructed to always tap on the square for one of the colors ("Go" condition) and to suppress their reaction for the other color ("No-Go" condition). The color rule remains the same throughout the entire experiment. Initially, there are ten practice trials. During the practice, participants receive feedback. The test round consists of 150 trials without feedback. 80% of the trials are "Go" stimuli. Participants have 750 ms to respond. The interval between stimuli varies between 250 and 750 ms.

Extracted Features

Variable Name	Description
goNogo_correct_mean_go	Average number of correct responses in "Go" trials.
goNogo_correct_mean_nogo	Average number of correct suppressions (no response) in "No-Go" trials.
goNogo_rt_mean_go	Average reaction time for responding in "Go" trials.
goNogo_rt_std_go	Standard deviation of reaction time in "Go" trials.

Bart

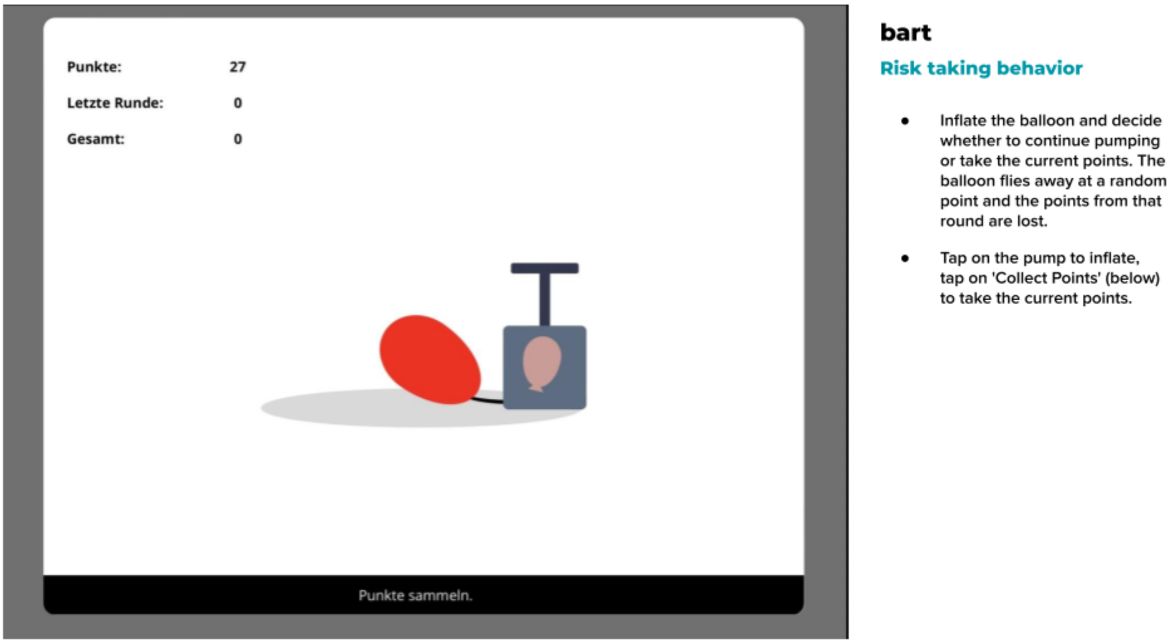

Participants see a balloon pump and a small balloon in the middle of the screen. At the bottom of the screen, there is a button labeled “Collect Points.” Participants are instructed to tap on the balloon pump to inflate the balloon. Each pump earns one point. At a random threshold that varies from trial to trial, the balloon flies away and the accumulated points are lost. Participants can end a round at any time by tapping the “Collect Points” button to secure the earned points, thus concluding the trial. The objective is to earn as many points as possible. After a practice round of four trials, the test round consists of 20 trials. The maximum number of pumps before the balloon flies away ranges from 1 to 50.

Extracted Features

Variable Name	Description
bart_correctedPumps	Number of pumps per trial adjusted for average number of pumps before the balloon would have burst
bart_changeAfterBurst	Changes in participant behavior following a balloon burst.
bart_didBurst	Indicator of whether the balloon burst in each trial.
bart_correctedTotal	Total adjusted score adjusted for average number of pumps before the balloon would have burst

n-Back

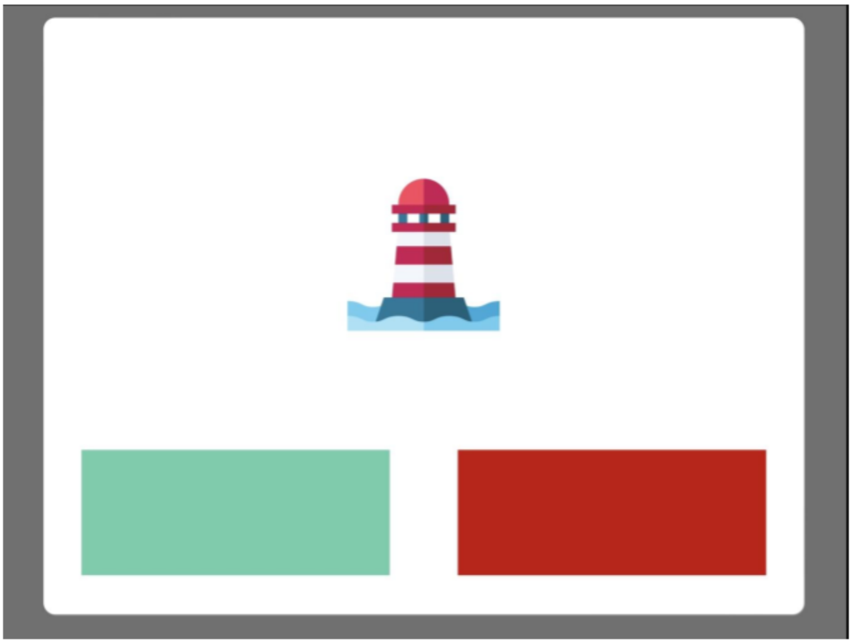

n-back

Working memory

- Is the current image the same as the one from one (or n) turns ago?
- Press the green button if the current image is the same as the previous one, press the red button if not.
For the first image, red is always correct (but it's not counted anyway). As the test progresses, the difficulty increases, n = 2: Is the current image the same as the penultimate image?

Participants see an image along with a green and a red button. They are instructed to tap the green button if the displayed image is the same as the one shown 1…n images before, and the red button if this is not the case. After a practice round of ten trials with feedback, there are two test rounds (n=1, n=2), each with 50 trials without feedback. The stimuli are displayed for 1200 ms, and participants have 3000 ms to respond."

Extracted Features

Variable Name	Description
NBack_correct_sum_load_1.0	Total number of correct responses for n-back level 1.
NBack_correct_sum_load_2.0	Total number of correct responses for n-back level 2.
NBack_rt_mean_load_1.0	Average reaction time for correct responses at n-back level 1.
NBack_rt_mean_load_2.0	Average reaction time for correct responses at n-back level 2.
NBack_rt_std_load_1.0	Standard deviation of reaction time for n-back level 1.
NBack_rt_std_load_2.0	Standard deviation of reaction time for n-back level 2.

Tower of London

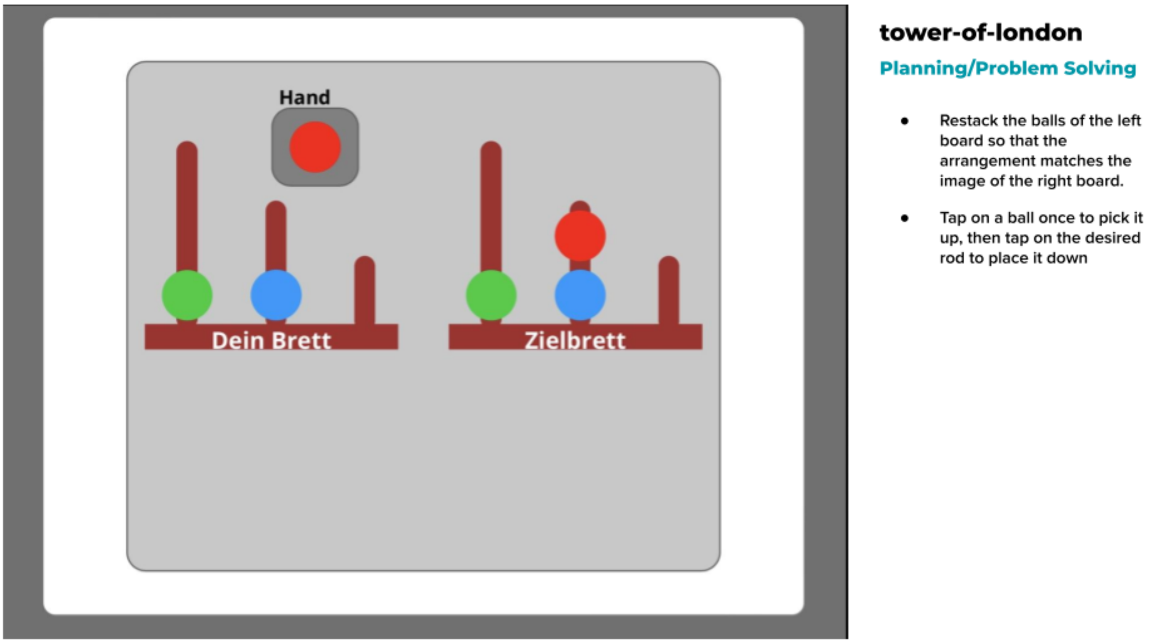

On each trial, participants were presented with two boards: their board and a target board. Each board contained three balls dispersed across three pegs.

Participants were instructed to make their board look like the target board by rearranging the colored balls while making as few moves as possible. Participants could move only one ball at a time and were instructed to plan their moves before execution. Each trial was capped at 20 seconds. Participants completed 12 trials of increasing difficulty (the optimal number of moves varied from 2 to 5).

Extracted Features

Variable Name	Description
TowerOfLondon_correct	Total number of correctly solved Tower of London problems.
TowerOfLondon_stim_duration	Time taken by the patient to complete each Tower of London trial.

Kirby

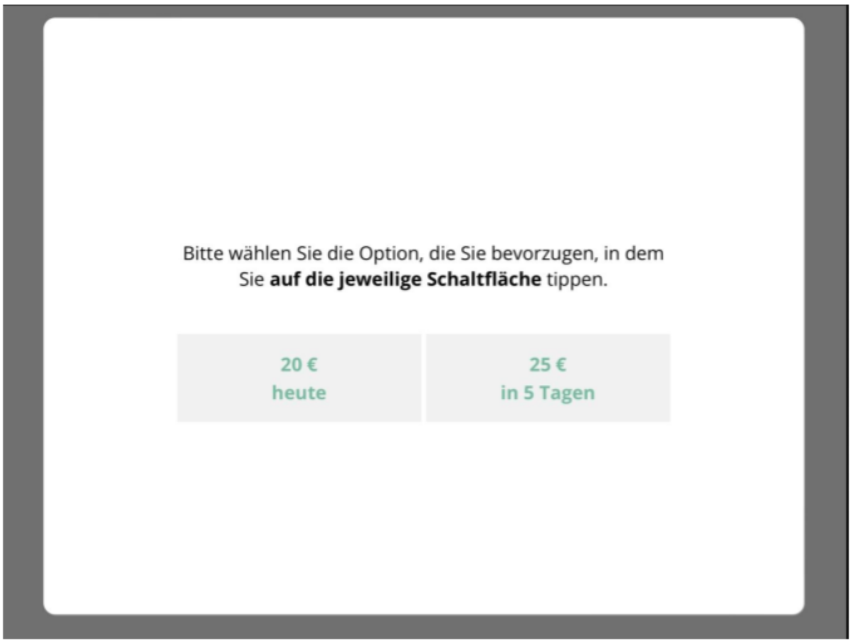

kirby

Delay discounting

- Decide whether to take a small amount of money immediately or a larger amount of money at a later time.

This is one of the most commonly used intertemporal choice tasks that is based on the multiple price list methodology in the economics literature. Similar to other intertemporal choice tasks in the battery, participants made choices between smaller immediate monetary amounts and larger delayed monetary amounts.
The stimuli were divided into three groups (small, medium, large), depending on the size of larger reward with nine choices in each group. Each of these nine choices spanned the same range of implied hyperbolic discount rates if they were to be the indifference points for a given participant (00.016-0.025) that were spaced equidistantly on a log-scale of hyperbolic discount rates.

Extracted Features

Variable Name	Description
kirby_expValue	Exponential discount rate value, indicating a consistent rate of discounting over time in the participant's choices.
kirby_hypValue	Hyperbolic discount rate value, reflecting a decreasing rate of discounting over time, typically leading to more impulsive decisions in the short term.

Flanker Modified for Children Task

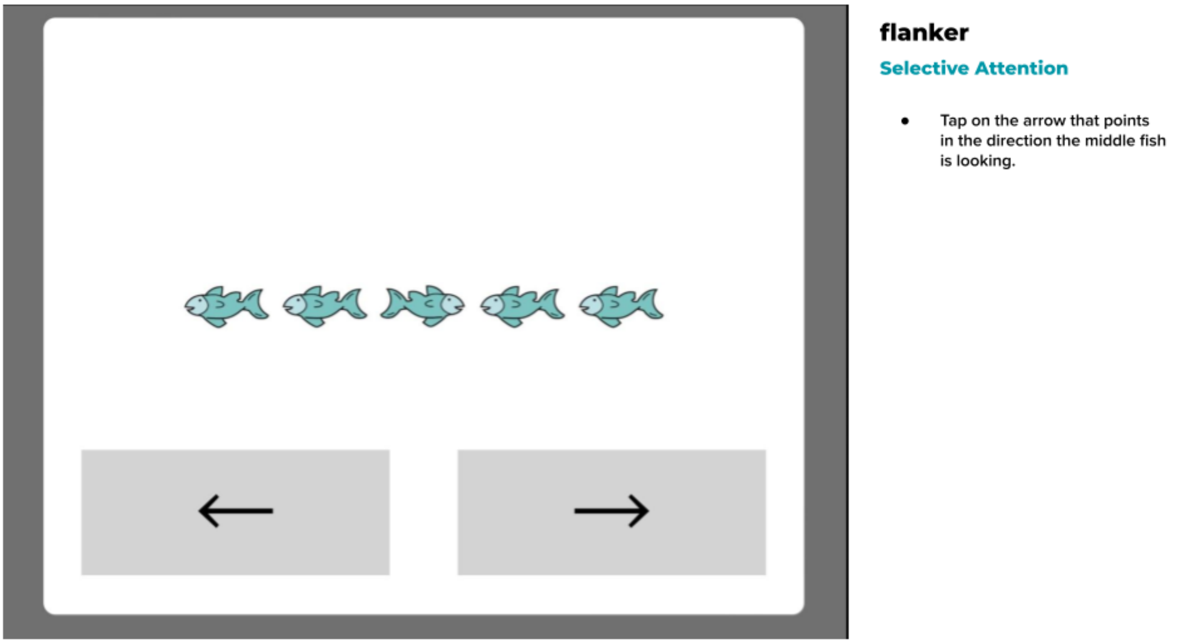

Participants see a row of five fish in the middle of the screen and two gray buttons at the bottom third of the screen. The left button shows an arrow pointing left and the right button shows an arrow pointing right. The fish either look in the same direction (congruent condition) or the gaze direction of the middle fish differs from the other fish (incongruent condition). In each trial, initially only the four flanking fish are displayed. After 100 ms, the middle fish appears and the timing begins. The buttons can only be used from this moment. Participants are instructed to tap the button whose arrow points in the direction the middle fish is looking. The fish are displayed for 3500 ms and participants can react during this time. After a practice round of ten trials with feedback, a test round of 30 trials without feedback follows.

Extracted Features

Variable Name	Description
flanker_rt_mean_congruent	Average reaction time for congruent trials.
flanker_rt_mean_incongruent	Average reaction time for incongruent trials.
flanker_rt_std_congruent	Standard deviation of reaction time in congruent trials.
flanker_rt_std_incongruent	Standard deviation of reaction time in incongruent trials.

Stop-Signal

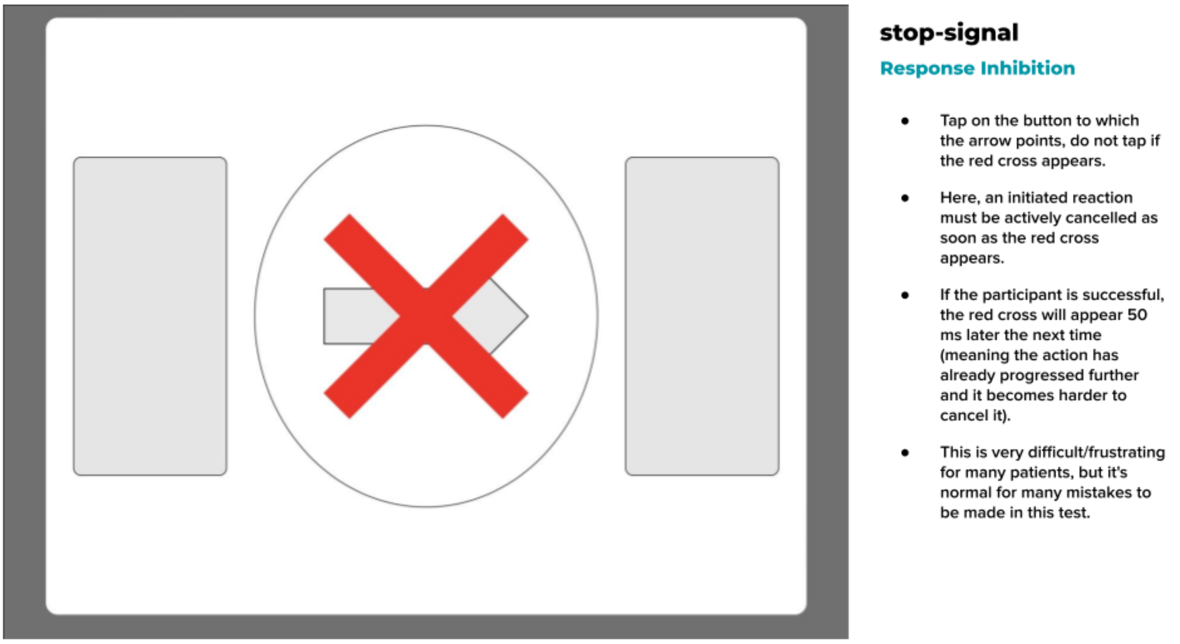

Participants see a circle in the middle of the screen with two buttons – one on the left and one on the right of the circle. In each trial, participants see an arrow inside the circle pointing either left or right. Participants are instructed to click on the button that the arrow points to. If a red cross appears, participants should suppress their reactions and not press any button. The red cross initially appears 250 ms after the arrow and dynamically adjusts based on the participant's performance: if the reaction is suppressed, the time increases by 50 ms, if not, the time decreases by 50 ms. The test has two conditions: “Low” and “High.” In the Low condition, one in five trials is a stop-signal, and in the High condition, it's two in five. After an extensive practice round of 30 trials with feedback, the practice may be repeated up to four times if performance is not fast enough or too many errors are made. After the practice, the test round consists of 50 trials per condition.

Extracted Features

Variable Name	Description
StopSignal_totalCorrect	Total number of correct responses across all trials.
StopSignal_correct_go	Number of correct responses in "go" trials.
StopSignal_correct_stop	Number of correct suppressions in "stop" trials.
StopSignal_max_value_delay_high	Maximum delay value for the stop signal in the High condition.
StopSignal_max_value_delay_low	Maximum delay value for the stop signal in the Low condition.

StopSignal_StopSignal_RT_mean_go	Average reaction time for "go" trials in stop-signal tests.
StopSignal_StopSignal_RT_std_go	Standard deviation of reaction time in "go" trials in stop-signal tests.

Reversal Learning

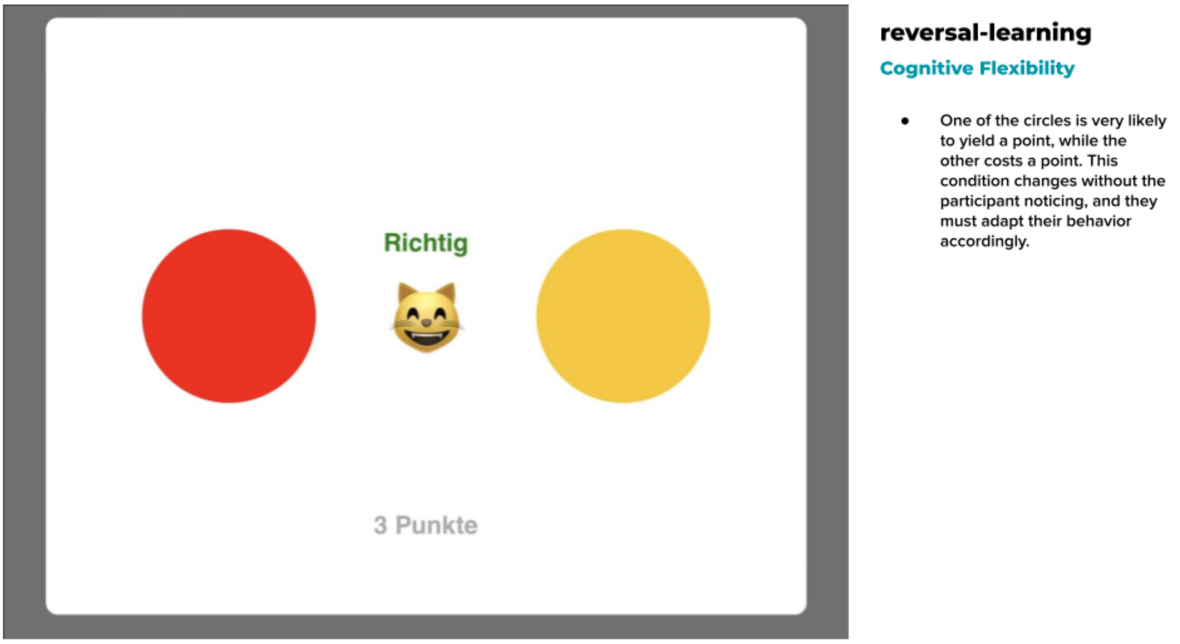

Participants see two differently colored circles. A point summary is displayed at the bottom of the screen. Participants are instructed to tap on one of the two circles. One of them usually results in a point gain, while the other typically leads to a point loss. At some point, the condition changes and the other color yields the point. Each block includes a condition change after 10-15 correct answers (including probabilistic errors). The number of probabilistic errors (i.e., 'incorrect' feedback) per condition varied between 0 and 4. To prevent participants from always changing their strategy after two consecutive error displays, each block included two consecutive trials with probabilistic negative feedback.

Extracted Features

Variable Name	Description
ReversalLearning_Correct	Total number of correct responses across all trials.
ReversalLearning_changeAfterProbabilisticWrongFeedback	Changes in participant behavior following probabilistic wrong feedback.
ReversalLearning_wasProbabilisticError	Indicator of whether a trial contained a probabilistic error.

ReversalLearning_reversalErrors	Number of errors made after a reversal in the task conditions.
ReversalLearning_probalisticErrors	Total number of probabilistic errors encountered across all trials.
ReversalLearning_totaltrials	Total number of trials completed in the task.
ReversalLearning_changeAfterProbabilisticWrongFeedbackRatio	Ratio of behavioral changes after probabilistic wrong feedback to total number of such feedback.

Judgment of line orientation task

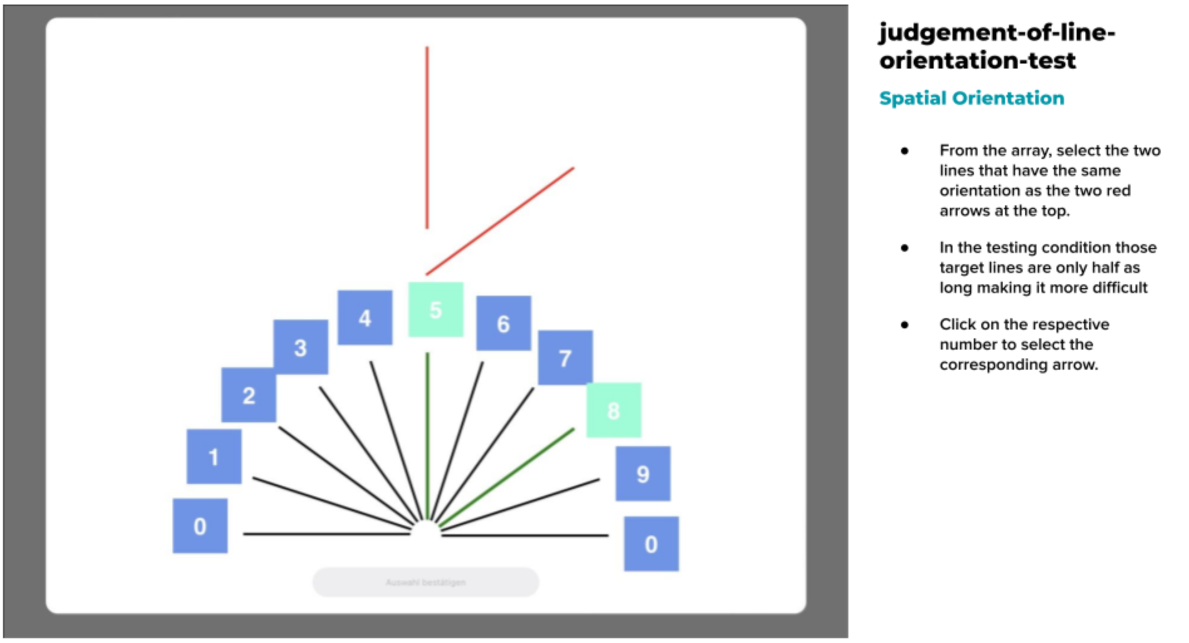

Participants see two red lines at the top of the screen, oriented differently. At the bottom, there is a 'fan' consisting of 11 lines, covering 180° in 18° steps. Each line has a button with a number from zero to nine (0° and 180° are treated as one condition). Participants are instructed to tap the buttons of the lines whose orientation corresponds to the two lines at the top. The input can be corrected until confirmed. Initially, a practice round of five trials is conducted, with participants receiving feedback. During the practice, the lines exactly match the length and spatial orientation of those depicted below. During the test rounds, only fragments of the original lines, one-third of their original length, are displayed at the top. In each trial, it randomly varies whether the left, middle, or right third of the original lines is displayed. The test consists of 30 trials.

Extracted Features

Variable Name	Description

LineOrientation_trial_time_mean	Average time taken by participants per trial to make their judgment.
LineOrientation_correct	Total number of correctly identified line orientations.
LineOrientation_distance	Measure of deviation from the correct orientation in participants' responses.

Information sampling

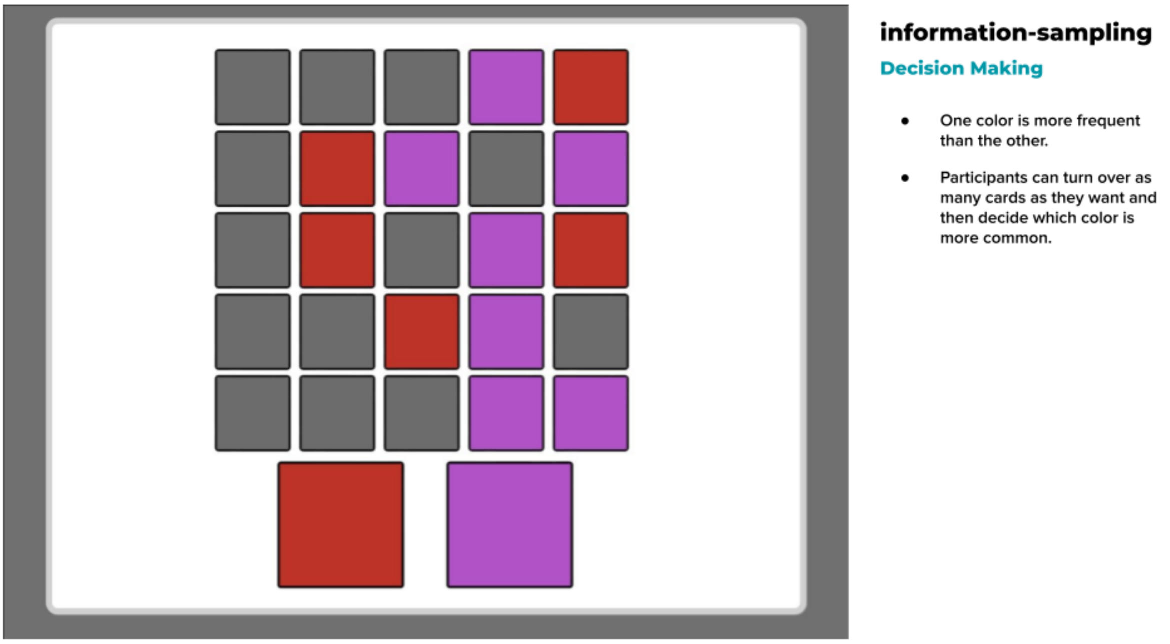

Participants were presented with a five by five grid of gray boxes where each box covered one of two colors. Participants were instructed to indicate which color they thought was the majority (one color made up between 13 and 18 of the boxes). To make this decision, participants were told they could reveal the color of any box by clicking on them. There were two conditions. In the fixed win condition, participants won or lost 100 points depending on the accuracy of their color choice regardless of how many boxes they opened. In the decreasing win condition, each round began with 250 points and each opened box cost 10 points on the potential winnings of the round. An incorrect choice in this condition also lead to a loss of 100 points.

Participants completed ten rounds of each condition. The DVs from this task were the average response latency of opening a box (motivation) and the average probability of making the correct decision in each round for each condition.

Extracted Features

Variable Name	Description
InformationSampling_rt_Decreasing Win	Average reaction time in trials with a decreasing win condition.
InformationSampling_rt_Fixed Win	Average reaction time in trials with a fixed win condition.
InformationSampling_which_click_in_round_Decreasing Win	Number of clicks within a round under the decreasing win condition.

InformationSampling_which_click_in_round_Fixed Win	Number of clicks within a round under the fixed win condition.
